# MoEnd3 regulates appressorium formation and virulence through mediating endocytosis in rice blast fungus *Magnaporthe oryzae*

**DOI:** 10.1371/journal.ppat.1006449

**Published:** 2017-06-19

**Authors:** Xiao Li, Chuyun Gao, Lianwei Li, Muxing Liu, Ziyi Yin, Haifeng Zhang, Xiaobo Zheng, Ping Wang, Zhengguang Zhang

**Affiliations:** 1 Department of Plant Pathology, College of Plant Protection, Nanjing Agricultural University, and Key Laboratory of Integrated Management of Crop Diseases and Pests, Ministry of Education, Nanjing, China; 2 Departments of Pediatrics, and Microbiology, Immunology, and Parasitology, Louisiana State University Health Sciences Center, New Orleans, Louisiana, United States of America; North Carolina State University, UNITED STATES

## Abstract

Eukaryotic cells respond to environmental stimuli when cell surface receptors are bound by environmental ligands. The binding initiates a signal transduction cascade that results in the appropriate intracellular responses. Studies have shown that endocytosis is critical for receptor internalization and signaling activation. In the rice blast fungus *Magnaporthe oryzae*, a non-canonical G-protein coupled receptor, Pth11, and membrane sensors MoMsb2 and MoSho1 are thought to function upstream of G-protein/cAMP signaling and the Pmk1 MAPK pathway to regulate appressorium formation and pathogenesis. However, little is known about how these receptors or sensors are internalized and transported into intracellular compartments. We found that the MoEnd3 protein is important for endocytic transport and that the Δ*Moend3* mutant exhibited defects in efficient internalization of Pth11 and MoSho1. The Δ*Moend3* mutant was also defective in Pmk1 phosphorylation, autophagy, appressorium formation and function. Intriguingly, restoring Pmk1 phosphorylation levels in Δ*Moend3* suppressed most of these defects. Moreover, we demonstrated that MoEnd3 is subject to regulation by MoArk1 through protein phosphorylation. We also found that MoEnd3 has additional functions in facilitating the secretion of effectors, including Avr-Pia and AvrPiz-t that suppress rice immunity. Taken together, our findings suggest that MoEnd3 plays a critical role in mediating receptor endocytosis that is critical for the signal transduction-regulated development and virulence of *M*. *oryzae*.

## Author summary

During the interaction between the rice blast fungus *Magnaporthe oryzae* and the host, the pathogen employs a series of receptors and sensors at the plasma membrane to recognize host surface cues and to activate signal transduction pathways required for appressorium formation and pathogenicity. We found that MoEnd3-mediated endocytosis is responsible for internalization of non-canonical GPCR Pth11 and the sensor MoSho1 to endosomal compartments. This is important for activating the downstream Pmk1 MAPK pathway to control appressorium formation and penetration. MoEnd3 is regulated through phosphorylation by the actin-regulating kinase MoArk1. In addition, MoEnd3 has a role in establishing effector secretion required for suppressing rice immunity. Our studies provide evidence that endocytosis is required for normal signaling and virulence in *M*. *oryzae*.

## Introduction

The rice blast fungus *Magnaporthe oryzae* produces an infectious structure called the appressorium that enables it to penetrate host plant cells and initiate infection [1]. During the interaction between the pathogen and the host, the fungus secretes numerous effectors into the host that suppress plant immunity [2–5]. Previous studies have shown that G-protein/cAMP signaling is important in the perception of host surface cues by *M*. *oryzae* and during invasion of host tissue [6, 7]. *M*. *oryzae* contains three distinct Gα subunit proteins: MagA, MagB and MagC as well as a highly conserved cAMP-dependent signaling pathway, which consists of the adenylate cyclase Mac1, the regulatory subunit of protein kinase A Sum1, and the catalytic subunit of protein kinase A cPKA [6, 8]. cPKA activation is responsible for appressorium differentiation. In addition, the non-canonical G-protein coupled receptor (GPCR) Pth11 is known to function upstream of G-protein/cAMP signaling [9, 10]. Moreover, the MAP kinase cascade comprised of Mst11 (MAPKKK), Mst7 (MAPKK), and Pmk1 (MAPK) is also involved in the regulation of appressorium formation [11]. Furthermore, MoMsb2 and MoSho1 function as two upstream sensors of the MAP kinase cascade [12]. Deletion of either *MoMSB2* or/and *MoSHO1* resulted in a significant reduction in appressorium formation. Intriguingly, the expression of a dominant active *MST7* allele partially suppressed the defects exhibited by the Δ*Momsb2* mutant [12].

Recently, endosomal compartments were discovered to function as signaling platforms by anchoring the components of G-protein/cAMP signaling. The various signaling components then interact within the endosomal compartments for sustaining signaling [13]. Endosomal compartments contain early and late endosomes. Proteins internalized from the cell surface target early endosomes to undergo a sorting process, by which they are either recycled back to the plasma membrane or sent to late endosomes for degradation. Previous studies have shown that disruption of phosphoinositide PI3P synthesis on the endosomal membrane or inhibition of the conversion of early endosomes into late endosomes by *MoVPS39* gene deletion disrupts the endosomal localization of Pth11, MagA, Mac1 proteins, and a regulator of G protein signaling MoRgs1 thereby leading to an inhibition in appressorium formation [13]. However, despite these important findings, the mechanism by which Pth11 or other receptors proteins enter intracellular compartments to activate signal transduction in *M*. *oryzae* is still unclear.

Endocytosis is a conserved intracellular transport process in which membrane proteins, lipids, or other macromolecules are transported to endosomal compartments. During endocytosis, endocytic proteins are recruited to endocytic sites and interact with actin cytoskeleton to drive vesicle maturation and scission [14]. In *Saccharomyces cerevisiae*, the Eps15 homolog (EH) domain-containing proteins Pan1p and End3p are important members of endocytic proteins and depletion of Pan1p or End3p severely impairs endocytosis and actin organization [15–17]. When vesicles are mature, endocytic proteins and actin components simultaneously dissociate from the vesicle membrane, thereby promoting efficient endocytosis [18]. The Ark1p/Prk1p actin-regulating kinases are implicated in this dissociation process [19, 20]. Ark1p/Prk1p phosphorylates Pan1p and other proteins to promote their dissociation [20, 21]. Deletion of Ark1p and Prk1p results in aggregation of endocytic proteins and actin cytoskeleton in the cytoplasm, which prevents endocytosis [22].

We previously found that MoArk1 has conserved functions in regulating endocytosis and that MoArk1 is required for appressorium turgor generation and penetration in *M*. *oryzae*. This study suggested that endocytosis plays an important role in the pathogenesis of the rice blast fungus [23]. Here we continued to investigate the mechanism that links MoArk1-regulated endocytosis to fungal pathogenesis. We identified a MoArk1-interacting protein MoEnd3 by mass spectrometry analysis and characterized its function. We found that MoEnd3 is an endocytic protein and mediates the endocytic transport of GPCR Pth11 and sensor MoSho1. This transport could trigger downstream Pmk1 phosphorylation for autophagy, appressorium formation and penetration. In addition, we identified that MoEnd3 function is regulated by MoArk1-dependent phosphorylation at Ser-222. Finally, we demonstrated that secretion of the MoEnd3-regulated effectors is directly linked to host immunity suppression.

## Results

### Identification of MoEnd3 as a MoArk1-interacting protein

MoArk1 is an actin-regulating kinase homolog required for endocytosis, growth, development, and full virulence of *M*. *oryzae* [23]. To explore the mechanism by which MoArk1 regulates these processes, we employed protein co-immunoprecipitation (Co-IP) to identify putative MoArk1-interacting proteins. By expressing the *MoARK1*:*FLAG* construct and using FLAG beads to isolate MoArk1:FLAG-interacting proteins followed by mass spectrometry analysis, we found several proteins potentially important for endocytosis and actin cytoskeleton, including homologues of the clathrin heavy chain, amylase-binding protein AbpA, Arp2/3 complex subunit proteins, endocytosis and cytoskeletal organization proteins, vesicular integral-membrane protein Vip36, and F-actin-capping proteins (S1 Table). Additional proteins co-precipitated with MoArk1 also include the dynamin-A homologue MoDnm1 that regulates peroxisomal and mitochondrial fission through interactions with MoFis1 and MoMdv1 [24].

**Fig 1:**
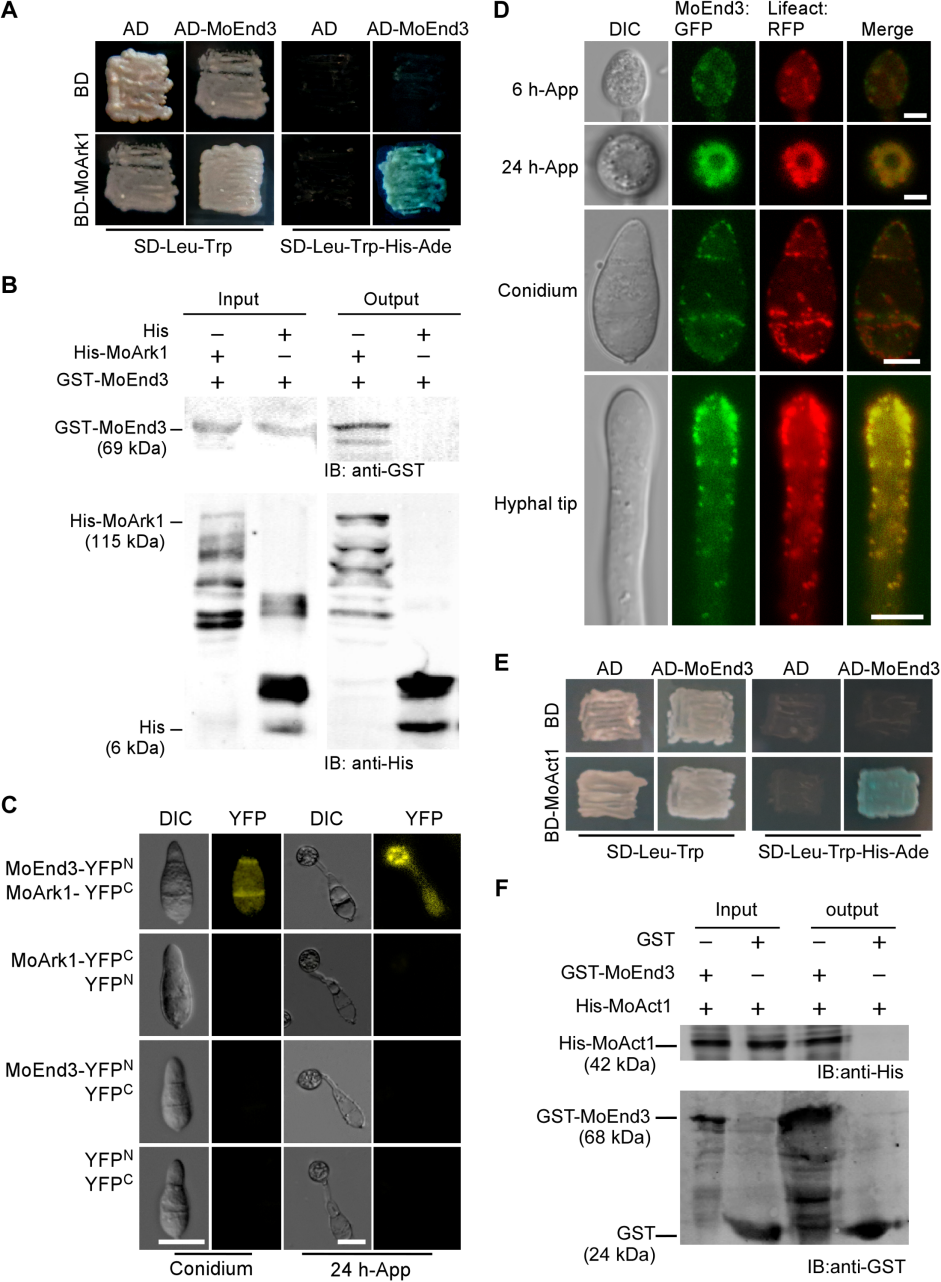
MoEnd3 interacts with MoArk1 and F-actin. (A) Yeast two-hybrid assay for examining the interaction between MoEnd3 and MoArk1. The yeast transformants were isolated from SD-Leu-Trp plates. Their β-galactosidase activity was assayed on SD-Leu-Trp-His-Ade plates containing X-Gal. The transformants expressing AD-MoEnd3 and empty BD, empty AD and BD-MoArk1, and empty AD and BD were used as negative control. (B) *In vitro* protein binding assay for the MoEnd3-MoArk1 interaction. Ni-NTA beads were used to bind His protein (6 kDa) as a negative control and His-tagged MoArk1 protein (115 kDa), respectively, and incubated with the GST-tagged MoEnd3 protein (69 kDa). The total proteins eluted from beads (output) were separated by 12% SDS-PAGE and immunoblotted with GST and His antibodies. (C) BiFC assay for the MoEnd3-MoArk1 interaction *in vivo*. Conidia and 24 h appressoria were examined by DIC and fluorescence microscopy. The strains expressing the MoArk1-YFP^C^ and empty YFP^N^, MoEnd3-YFP^N^ and empty YFP^C^, empty YFP^N^ and empty YFP^C^ constructs were used as negative controls, Bars = 10 μm. (D) MoEnd3:GFP is co-localized with F-actin. The localization pattern of MoEnd3:GFP was displayed in 6 h and 24 h-appressoria, conidium and hyphal tip region. F-actin was labeled with Lifeact:RFP. Bars = 10 μm. (E) Yeast two-hybrid assay was used to examine the interaction between MoEnd3 and actin. Transformants were isolated from SD-Leu-Trp plates and their β-galactosidase activity was assayed on SD-Leu-Trp-His-Ade plates containing X-Gal. Transformants expressing AD and BD, BD-MoAct1 and AD, and BD and AD-MoEnd3 were used as negative controls. (F) Protein binding assay for MoEnd3-MoAct1 interaction *in vitro*. GST-beads were used to bind GST protein (24 kDa) or GST-tagged MoEnd3 protein (68 kDa), respectively, and incubated with His-tagged MoAct1 protein (42 kDa). Total eluted fractions from the beads (output) were immunoblotted with the His and GST antibodies. MoEnd3 is important for sexual reproduction and normal endocytosis.

We identified MGG_06180.6 as an endocytic protein homolog to *S*. *cerevisiae* End3p (30% amino acid sequence identity) and characterized its function. To confirm the interaction between MoEnd3 and MoArk1, we employed the yeast two-hybrid assay that demonstrated the interaction. Transformants expressing AD-MoEnd3 and BD-MoArk1 constructs showed β-galactosidase activity on SD-Leu-Trp-His-Ade plates (Fig 1A). In addition, we performed *in vitro* protein binding and bimolecular fluorescence complementation (BiFC) assays that further substantiated the MoEnd3 and MoArk1 interaction (Fig 1B and 1C). In the BiFC assay, fluorescence appeared in the cytoplasm of the conidia and 24 h appressorium of the strain co-expressing MoEnd3-YFP^N^ and MoArk1-YFP^C^ constructs, but not in controls (Fig 1C).

To characterize MoEnd3 functions, a Δ*Moend3* mutant was obtained (S1 Fig) and characterized. No significant differences were observed between the Δ*Moend3* mutant and the wild-type Guy11 strain in colony diameter (on CM, MM, SDC and OM medium plates) or conidia production (S2 Table). However, when the Δ*Moend3* mutant was crossed to the tester strain TH3 (MAT1-1), no perithecia were observed after 3 weeks (S2 Fig), suggesting that MoEnd3 is dispensable for vegetative growth and conidiation but not sexual reproduction.

**Fig 2:**
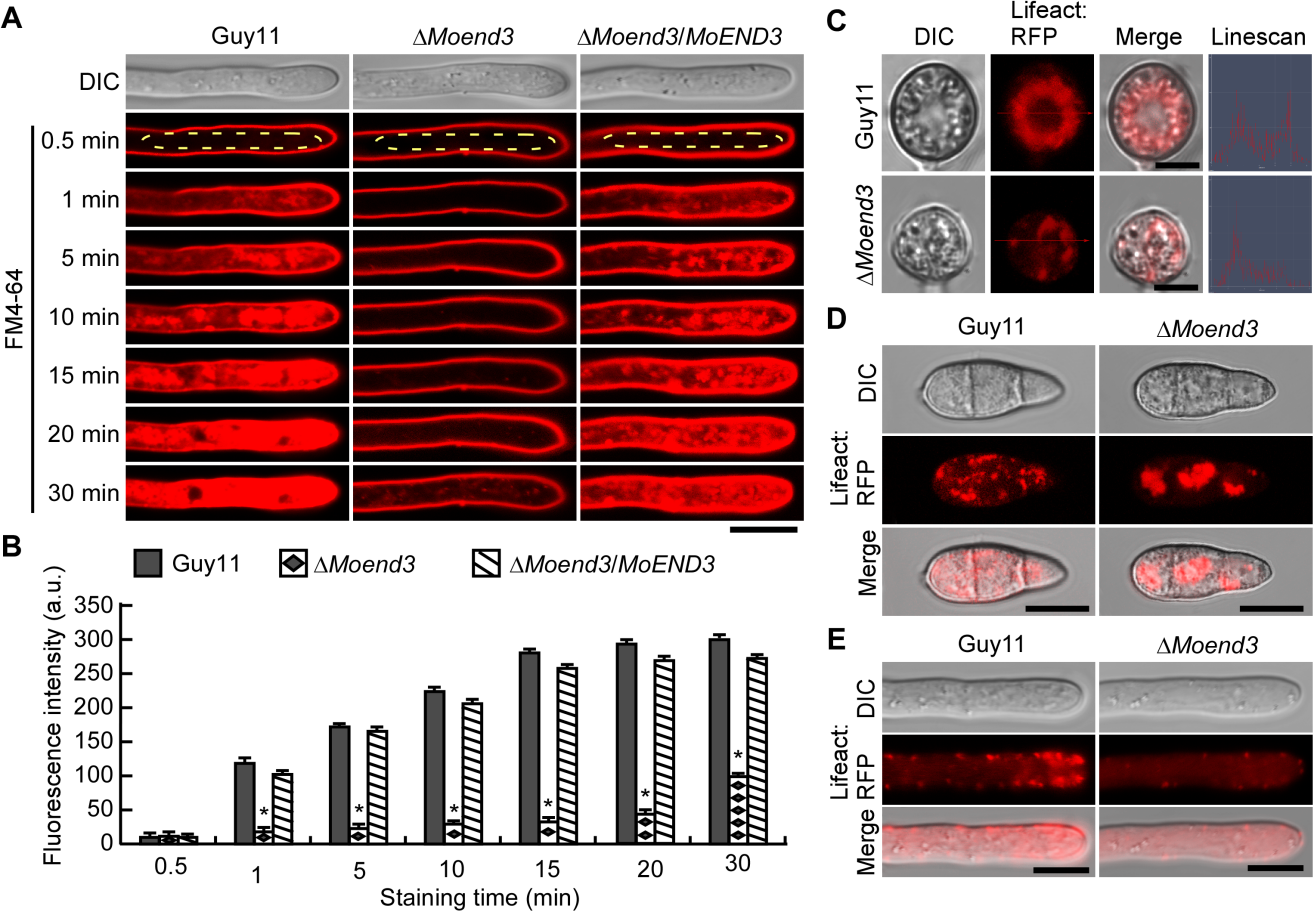
MoEnd3 is involved in endocytosis and F-actin assembly. (A) Time course-images of FM4-64 uptake at the hyphal tips. Hyphae stained by FM4-64 were examined by using fluorescence microscopy at different time points. The regions where fluorescence intensity was measured by ImageJ software were labeled by ellipse frame. Bars = 5 μm. (B) The bar chart shows the mean fluorescence intensity at the hyphal tip region. At least 15 hyphae of each strain were measured by applying ImageJ software at each time point. Error bars represent standard deviation (SD) and asterisks represent significant differences (P < 0.01). a.u., arbitrary unites. (C) F-actin network in appressoria (24 h) of Guy11 and Δ*Moend3*. Line-scan graphs show Lifeact:RFP fluorescence in a transverse section of individual appressorium. Bars = 5 μm. (D) F-actin in conidia of Guy11 and Δ*Moend3*. Bars = 10 μm. (E) Actin patches in hyphal tip regions. Bars = 10 μm.

To examine whether MoEnd3 is required for endocytosis, we stained the cells with the lipophilic dye FM4-64 and observed its internalization. After 1 min of staining, the dye appeared in the cytoplasm of hyphal tips in Guy11 and the complemented strain, but the dye remained at the plasma membrane of the Δ*Moend3* mutant (Fig 2A). At 15 min, the dye was most intense in the hypal tip of Guy11 and the complemented strain, while was near invisible in the cytoplasm of Δ*Moend3*. Only at 30 min, when some dye internalization was observed in Δ*Moend3*. The fluorescence intensity of the dye was quantified using the ImageJ software (Fig 2B), and this quantification is consistent in suggesting that MoEnd3 is required for normal endocytosis.

### MoEnd3 is involved in F-actin assembly

Since the End3 endocytic protein regulates endocytosis through the coordination of the F-actin assembly at endocytic sites in *S*. *cerevisiae* [25], we examined whether *MoEND3* deletion impairs F-actin organization using the Lifeact:RFP marker [26]. A toroidal-shaped F-actin network could be observed in 80.4% of the mature appressoria produced by wild-type Guy11 (Fig 2C). By comparison, Δ*Moend3* displayed an aberrant distribution of F-actin in 98.8% of appressoria, as demonstrated by a line-scan analysis. It is known that the actin patch that associates with plasma membrane corresponds to endocytic sites [27]. In the conidia of Guy11, a lot of punctae-like cortical actin patches were observed in the cytoplasm of conidia (Fig 2D). However, aggregated, instead of punctae-like, actin structures were observed in nearly 96.3% of Δ*Moend3* conidia (Fig 2D). In addition, many actin patches displayed polarized distributions at the hyphal tip regions of Guy11, whereas they were rarely seen at the hyphal tip region of Δ*Moend3* (Fig 2E).

To further examine whether MoEnd3 is associated with F-actin, the MoEnd3:GFP fusion protein and Lifeact:RFP were co-expressed in the Δ*Moend3* mutant and localizations of the GFP and RFP fusion proteins were observed by confocal fluorescence microscopy. We found that MoEnd3:GFP co-localized with the F-actin network in appressoria after 6 and 12 h of incubation (Fig 1D). In conidia and the hyphal tips, MoEnd3:GFP patches were found at the plasma membrane and were co-localized with actin patches (Fig 1D). However, we still observed some regions only showed MoEnd3:GFP or Lifeact:RFP, likely due to that End3 protein arrives endocytic sites or disassembles from there earlier than F-actin, as suggested in studies involving *S*. *cerevisiae* End3p [16, 27].

We then examined whether MoEnd3 interacts with F-actin protein MoAct1 by performing yeast two-hybrid and *in vitro* protein binding assays. Consistently, both assays demonstrated an interaction occurred between MoEnd3 and MoAct1 (Fig 1E and 1F), supporting that MoEnd3 could coordinate actin assembly through a direct interaction with F-actin.

### MoEnd3 affects appressorium formation and virulence

**Fig 3:**
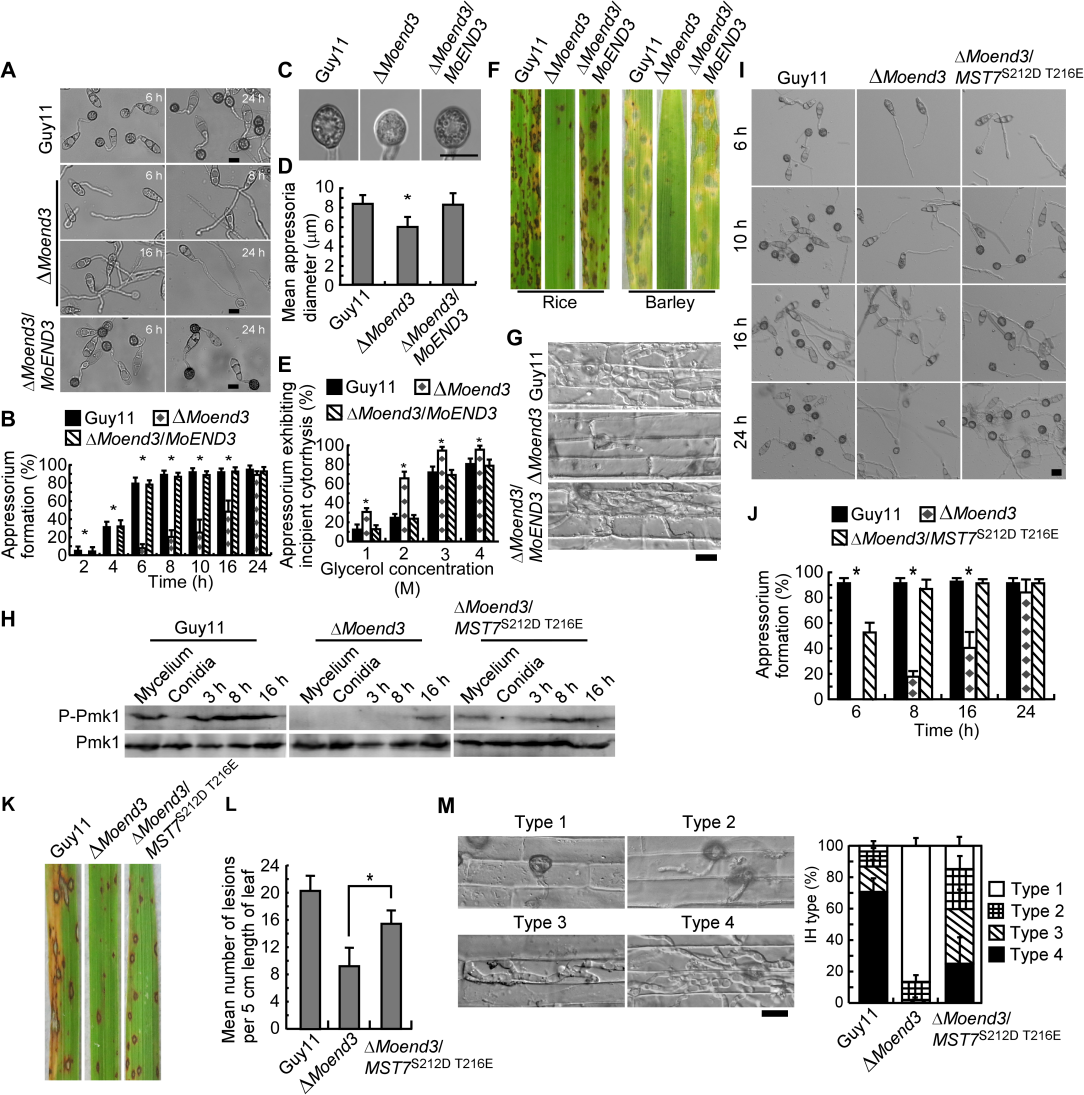
MoEnd3 is important for appressorium formation and virulence. (A) Appressorium formation assay. Conidia were incubated on hydrophobic surfaces and the samples were observed at different time points. Bar = 10 μm. (B) Appressorium formation rates at different time points were calculated and statistically analyzed. The percentage at a given time was recorded by observing at least 200 conidia for each strain and the experiment was repeated three times. Error bars represent SD and asterisks represent significant differences (P < 0.01). (C) Images show appressoria after 24 h incubation on hydrophobic surfaces. Bar = 10 μm. (D) Mean appressorium diameter. The values were recorded by observing at least 100 appressoria for each strain and the experiment was repeated three times. Error bars represent SD and asterisk represents significant difference (P < 0.01). (E) Appressorium turgor was measured by an incipient cytorrhysis (cell collapse) assay. The percentage of collapsed appressoria was recorded by observing at least 100 appressoria and the experiment was repeated three times. Error bars represent SD and asterisks represent significant differences (P< 0.01). (F) Conidial suspensions of strains were sprayed onto 2-week old rice seedlings (CO-39) and 7-day old barley. Diseased rice and barley leaves were photographed after 7 and 5 days of inoculation, respectively. (G) Penetration assay with rice sheath. Excised rice sheath from 4-week-old rice seedlings was inoculated with conidial suspension. Images show invasive growth in rice sheath epidermal cells at 36 hpi. Bar = 10 μm. **(**H) Pmk1 phosphorylation level analysis with proteins extracted from mycelium, conidia, and conidia or appressoria incubated on hydrophobic surfaces for 3 h, 8 h and 16 h. The phosphorylation levels of Pmk1 (42-kDa) were detected using a phosphor-MAPK antibody (upper panel). The endogenous Pmk1 was detected using a MAPK antibody (lower panel). (I) Appressorium formation assay on the hydrophobic surfaces. (J) Appressorium formation rates were calculated and statistically analyzed. Asterisks represent significant differences (P<0.01). (K) Pathogenicity assay on rice (CO-39). (L) Quantification of the lesion numbers per 5 cm length of rice leaf. Error bars represent SD and asterisk represents significant difference (P<0.01). (M) Penetration assays in rice sheath. IH growth on rice cells was observed at 36 hpi and 4 types of IH were quantified and statistically analyzed. Error bars represent SD. Micrographs show 4 types of IH in rice cells. Bar = 10 μm. MoEnd3 is involved in endocytosis of Pth11 and MoSho1.

On hydrophobic surfaces, the Δ*Moend3* mutant showed delayed appressorium development compared with Guy11 and the complemented strain (Fig 3A and 3B) and this delay became indistinguishable after 24 h. However, the germ tubes of Δ*Moend3* were elongated and the appressoria were smaller in size and not fully developed (Fig 3C and 3D). The incipient collapse assay [28] showed that the collapse rate of appressoria of Δ*Moend3* was significantly higher than Guy11 and the complemented strain (Fig 3E), suggesting that MoEnd3 contributes to appressorial turgor generation.

We further observed translocation and degradation of glycogen and lipid required for turgor generation during conidia germination and appressoria development. Iodine solution and Nile red were used to stain the glycogen and lipid bodies, respectively. At 0 h, the glycogen and lipids were abundant in conidia (S3 Fig). In Guy11, the glycogen and lipids were translocated from conidia to nascent appressoria and were rapidly degraded in conidia after 6 h. They were completely degraded in over 60% of conidia after 12 h and in 90% of the mature appressoria after 24 h. In Δ*Moend3*, the degradation of glycogen in conidia and its translocation to appressoria occurred more slowly, and this was coupled with the delayed appressorium formation. After 12 h, glycogen and lipids in conidia were not translocated or degraded. After 24 h, they remained in almost 50% of conidia. These results suggested that MoEnd3 is required for an efficient translocation and breakdown of glycogen and lipids.

To further test the role of MoEnd3 in pathogenesis, conidial suspensions were sprayed onto susceptible rice seedlings (*Oryza sativa* cv. CO-39). After 7 days of inoculation, Δ*Moend3* produced significantly fewer lesions than control strains. The lesions produced by Δ*Moend3* were also smaller and less expansive, in contrast to the fully expanded necrotic lesions produced by Guy11 and the complemented strain (Fig 3F). Similar results were obtained in barley leaf infection assay after 5 days (Fig 3F). To further validate the reduction in virulence of Δ*Moend3*, we performed penetration assays using detached barley leaf. By observing 100 appressoria for each strain at 24 hpi and classifying their invasive hyphae (IH) into 4 types (type 1, no hyphal penetration; type 2, IH with one or two branch; type 3, IH with at least three branch, but the IH are short and less extended; type 4, IH that has numerous branches and fully occupies a plant cell), we found that in Guy11 and the complemented strain, nearly 80% of appressoria were type 3, in contrast to that 52.3% were type 1 and 38.1% were type 2 in Δ*Moend3* (S4 Fig). In the penetration assays using rice tissues, 90.2% of appressoria of Guy11 and the complemented strain displayed extended IH growth, whereas less than 10% of Δ*Moend3* appressoria formed IH, which were arrested in individual rice cells and did not extend to neighboring cells (Fig 3G). These results indicated that MoEnd3 is required for full virulence.

Pth11 is a non-canonical GPCR that functions upstream of the G-protein/cAMP pathway for surface sensing in *M*. *oryzae* [9]. Once proper surface clues were sensed by *M*. *oryzae*, Pth11 and cAMP signaling components, such as MagA and MoRgs1, are anchored on the endosomal compartments to sustain the transduction of cAMP signaling [13]. In addition, membrane sensors MoMsb2 and MoSho1 are responsible for recognition of surface signals and activation of the downstream MAPK cascade consisting of Mst11-Mst7-Pmk1 [12]. Both the cAMP pathway and the Pmk1-MAPK cascade are known to regulate appressorium formation and penetration.

**Fig 4:**
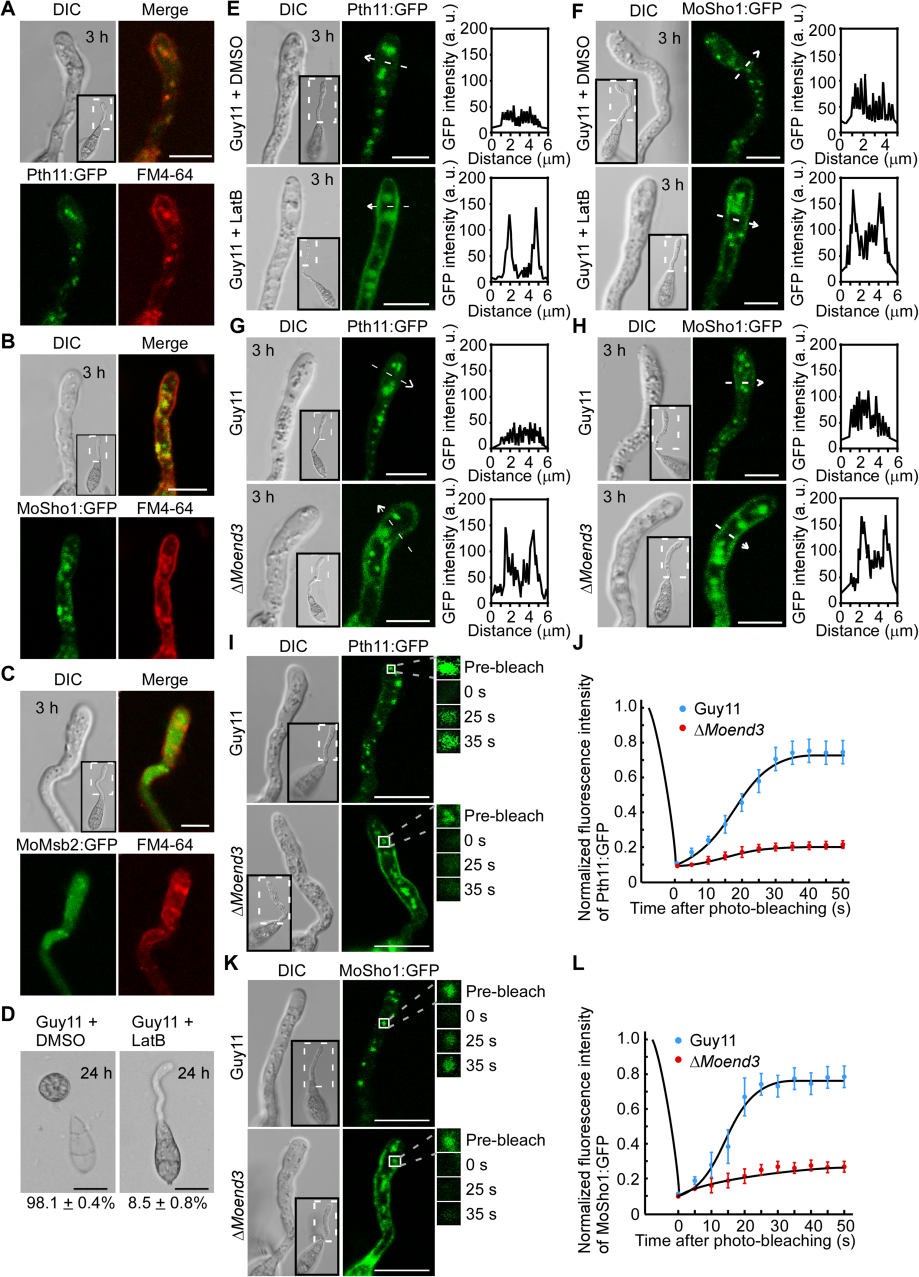
Pth11 and MoSho1 are transported by MoEnd3-mediated endocytosis. (A) Pth11:GFP was co-localized with FM4-64 in cytoplasm of germ tubes at 3 h. Merged image shows the GFP channel and FM4-64. Bar = 5 μm. (B) MoSho1:GFP was co-localized with FM4-64 in cytoplasm of germ tubes at 3 h. Merged image shows the GFP channel and FM4-64. Bar = 5 μm. (C) MoMsb2:GFP punctuate structures were not co-localized with FM4-64 marked endosomes in germ tubes. Merged image shows the GFP channel and FM4-64. Bar = 5 μm. (D) Appressorium formation assay with addition of LatB. Guy11 conidia significantly reduced appressorium formation after exposure to LatB for 30 min. The appressorium formation rates were recorded by observing 100 conidia for each sample and the experiment was repeated three times. (E) Pth11:GFP localization patterns with DMSO solvent and LatB treatment in germ tubes of Guy11 at 3 h. Insets highlight areas analyzed by line-scan. Bars = 5 μm. (F) MoSho1:GFP localization patterns with DMSO solvent and LatB treatment in germ tubes of Guy11 at 3 h. Insets highlight areas analyzed by line-scan. Bars = 5μm. (G) Pth11:GFP localization pattern in germ tubes of Guy11 and Δ*Moend3* at 3 h. Insets highlight areas analyzed by line-scan. Bars = 5 μm. (H) MoSho1:GFP localization pattern in germ tubes of Guy11 and Δ*Moend3* at 3 h. Insets highlight areas analyzed by line-scan. (I) Representative images of FRAP analysis for diffusion at Pth11:GFP localized regions in germ tubes of Guy11 and Δ*Moend3*. The fluorescence of Pth11:GFP significantly recovered at 35 s post-photobleaching in Guy11 but not in Δ*Moend3*. (J) Normalized FRAP curves of Pth11:GFP localized regions in Guy11 and Δ*Moend3*. 20 regions from different cells were subjected to FRAP analysis for each strain. Intervals: 5 s. (K) Representative images of FRAP analysis for diffusion at MoSho1:GFP localized regions in germ tubes of Guy11 and Δ*Moend3*. The fluorescence of MoSho1:GFP significantly recovered at 35 s post-photobleaching in Guy11 but not in Δ*Moend3*. (L) Normalized FRAP curves of MoSho1:GFP localized regions in Guy11 and Δ*Moend3*. 20 regions from different cells were subjected to FRAP analysis for each strain. Intervals: 5 s.

In mammalian cells, endocytosis transports membrane receptors or sensors to endosomes so that these receptors and sensors interact with signaling proteins to activate and amplify signal transduction [29]. We examined whether Pth11, MoMsb2, and MoSho1 are transported by endocytosis. We expressed Pth11:GFP, MoMsb2:GFP, and MoSho1:GFP in Guy11 and observed their co-localization with FM4-64 in germ tubes following conidia incubation on hydrophobic surfaces for 3 h. This stage is crucial for pathogen to sense surface clues and initiate appressorium development. We observed that signal of Pth11:GFP and MoSho1:GFP, but not MoMsb2:GFP, was primarily accumulated in regions also labeled by FM4-64 (Fig 4A, 4B and 4C).

Rab5 GTPase and Rab7 GTPase are known to bind with early endosomes and late endosomes, respectively [30]. To determine whether FM4-64 stained regions in germ tubes are endosomes or vacuoles, co-localizations of FM4-64 with GFP:Rab5 or GFP:Rab7 and vacuole marker CMAC were observed in germ tubes (S5 Fig). We found that most of FM4-64 was localized to GFP:Rab5 labeled regions (S5A Fig) and rarely co-localized with GFP:Rab7 (S5B Fig). In addition, CMAC-marked vacuoles did not appear in the germ tubes but only in the conidia. These observations revealed that internalized FM4-64 localizations in germ tube are likely to be early endosomes. Considering our finding that Pth11:GFP and MoSho1:GFP were co-localized with FM4-64, we proposed that most of Pth11 and MoSho1 are localized to early endosomes of the germ tubes.

To further demonstrate that Pth11 and MoSho1 are internalized by endocytosis, we used actin inhibitor Latrunculin B (LatB) that inhibits endocytosis [14] and determined the effect of LatB on Pth11 and MoSho1. We found that LatB inhibited Pth11:GFP and MoSho1:GFP internalization and enriched them at plasma membrane (Fig 4E and 4F). In addition, exposure to Lat B for 30 min resulted in 91.5% of germinated conidia being unable to form appressorium (Fig 4D).

Next we determined the role of MoEnd3 in endocytosis of Pth11:GFP and MoSho1:GFP. We found that most of the Pth11:GFP and MoSho1:GFP signals remained at the plasma membrane of the germ tubes in Δ*Moend3* (Fig 4G and 4H), and this pattern is similar to that of Pth11:GFP and MoSho1:GFP in Guy11 treated with LatB. We further compared Δ*Moend3* and Guy11 in the endocytosis rate of Pth11 and MoSho1 by fluorescence recovery after photobleaching (FRAP), a technique that measures the mobility of fluorescent proteins. We intended to bleach fluorescence from the regions where Pth11:GFP or MoSho1:GFP were accumulated in germ tubes and the recovery of fluorescence can reflect the rate of endocytosis. Considering newly synthesized proteins can be delivered from Golgi to endosomes, we treated germinated conidia (3 h) with cycloheximide to inhibit protein biosynthesis, which may prevent Golgi resident Pth11:GFP or MoSho1:GFP from entering endosomes. We also treated germinated conidia with benomyl for 10 min to inhibit endosomes trafficking via microtubule [31, 32]. In the FRAP assay, we bleached 90% of fluorescence of a region using 488 nm light. For Pth11:GFP, 72.7 ± 4% of fluorescence was recovered at post-photobleach 35 s in Guy11, compared with 16.1 ± 0.8% in Δ*Moend3* (Fig 4I and 4J). In addition, the recovery level of MoSho1:GFP in Δ*Moend3* (27.5 ± 3.1%) was significantly lower than that in Guy11 (78.8 ± 7.9%) at post-photobleach (Fig 4K and 4L). Collectively, these results suggested that MoEnd3 is important for endocytosis of Pth11 and MoSho1.

### MoEnd3 contributes to Pmk1 phosphorylation

It is clear that the Mst11-Mst7-Pmk1 MAPK pathway is required for appressorium formation and function [11]. Since Δ*Moend3* showed defects in appressorium formation, penetration and endocytosis of Pth11 and MoSho1, we tested the hypothesis that Mst11-Mst7-Pmk1 signaling could also be affected in Δ*Moend3*. We extracted proteins and performed Western blot analysis and found that there was no difference in the expression of Pmk1 (42-kDa) between Δ*Moend3* and Guy11 (Fig 3H bottom panel). By using the phosphor-MAPK antibody, Pmk1 phosphorylation was detected at all stages except conidia in Guy11 (Fig 3H bottom panel). However, a reduced Pmk1 phosphorylation level was detected in the Δ*Moend3* appressoria following 16 h of incubation. This finding suggested that MoEnd3 affects Pmk1 phosphorylation during appressorium development.

Previous studies showed that the constitutively activated *MST7*^S212D T216E^ allele restores normal Pmk1 phosphorylation and appressorium formation in the Δ*mst11* and Δ*mst7* mutant strains [11]. To confirm that MoEnd3 affects Pmk1 phosphorylation, we introduced the *MST7*^S212D T216E^ allele into Δ*Moend3* and found that it too suppressed the defect of Δ*Moend3* in appressorium formation (Fig 3H upper panel). Interestingly, 50% of conidia of the Δ*Moend3*/*MST7*^S212D T216E^ strain appeared to form appressoria after 6 h of incubation on hydrophobic surfaces, whereas no appressoria were formed in Δ*Moend3*. There were no significant differences in the formation rate between Guy11 and the Δ*Moend3*/*MST7*^S212D T216E^ strain after 10 h (Fig 3I and 3J). Moreover, Δ*Moend3* only formed a small number of lesions on rice leaves (Fig 3K and 3L). In contrast, the Δ*Moend3*/*MST7*^S212D T216E^ strain produced many typical lesions (Fig 3K and 3L). Further, penetration assays using rice tissues were conducted by observing 100 appressoria for each strain and classifying their IH into 4 types (type 1, no hyphal penetration; type 2, IH with less than two branches; type 3, IH with at least two branches, but the IH are short and less extended; type 4, IH that fully occupies a plant cell and moves into neighboring cells). We found that 84.2% of appressoria from the Δ*Moend3*/*MST7*^S212D T216E^ strain could penetrate the rice cells (Fig 3M). In contrast, less than 10% of appressoria from Δ*Moend3* could penetrate the host. These results suggested a function link between MoEnd3 and Pmk1 by showing that elevating Pmk1 phosphorylation level could significantly suppress the defect of Δ*Moend3* in appressorium formation and infection.

### MoEnd3 is important for autophagy

**Fig 5:**
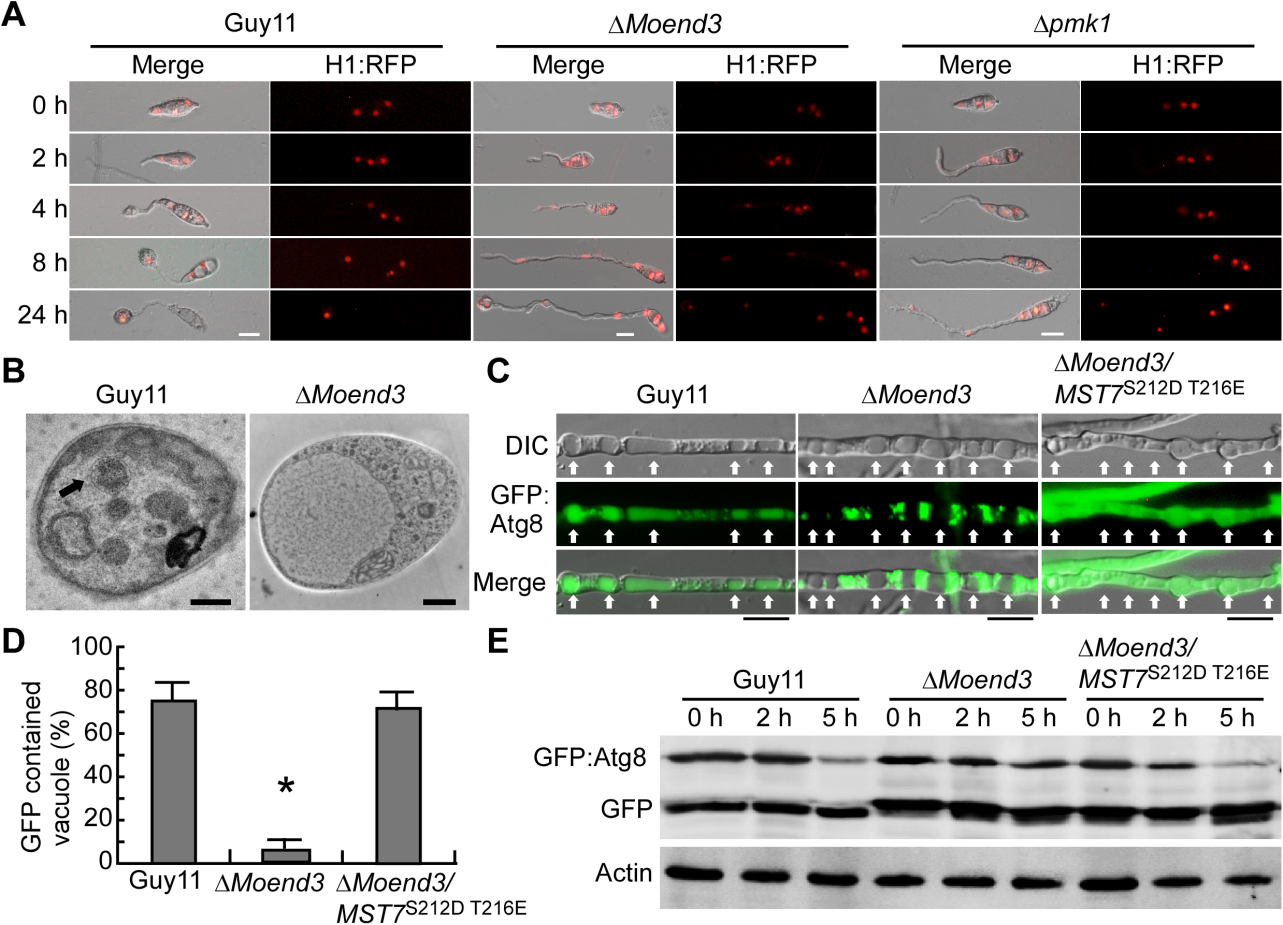
MoEnd3 functions in nuclear degradation and autophagy. (A) Nuclei were visible during appressorium development using H1:RFP. The merged image shows H1:RFP and DIC. Bars = 10 μm. (B) Electron micrographs of vacuoles in hyphae following 4 h nitrogen starvation condition. Arrows indicate autophagosomes. Bars = 0.5μm. (C) Hyphae from the strains expressed GFP:MoAtg8 were exposed to nitrogen starvation for 4 h in the presence of 4 mM PMSF. Arrows indicate vacuoles. Merged image shows the GFP:MoAtg8 and DIC. Bar = 10μm. (D) The percentages of vacuoles containing GFP:MoAtg8 were recorded by observing at least 100 vacuoles for each sample, and the experiment was repeated three times. Error bars represent SD and asterisk represents significant difference (P < 0.01). (E) GFP:MoAtg8 proteolysis assay. Total proteins were extracted from the GFP:MoAtg8 expressed strains exposed to nitrogen starvation condition for 0, 2 and 5 h. The full-length GFP:MoAtg8 and free GFP were detected using GFP antibodies. Protein contents were analyzed using the actin antibody.

Nuclear degradation in conidia is essential for appressorium development and penetration, which is also the consequence of autophagy following mitosis and nuclear migration [33]. To test if MoEnd3 has a role in autophagy, an RFP-labeled H1 histone protein (H1:RFP) was expressed in both Guy11 and the Δ*Moend3* mutant, and nuclei were visualized following conidia germination on the hydrophobic surface. Δ*Moend3* displayed successive nuclear divisions, with no breakdown of nuclei in conidia or germ tubes at 24 h (Fig 5A). We also expressed H1:RFP in the Δ*pmk1* mutant and found that nuclei failed to degrade (Fig 5A), consistent with previous study [33]. Thus, it is likely that the defect in nuclear degradation in Δ*Moend3* is due to the defective Pmk1 phosphorylation.

We then determined whether deletion of *MoEND3* affects autophagy by culturing mycelia in liquid minimal medium with reduced nitrogen (MM-N) in the presence of the proteinase B inhibitor phenylmethylsulfonyl fluoride (PMSF) for 4 h and observing hyphal vacuoles under a electron microscope. Autophagosomes were observed in the vacuoles of Guy11 but not Δ*Moend3* (Fig 5B). The *GFP*:*MoATG8* construct can be used as a functional marker for monitoring the delivery of vesicles to vacuoles and the breakdown of autophagosomes, and normal autophagy cannot easily hydrolyze free GFP protein cleaved from GFP:MoAtg8 [24, 34, 35]. We monitored autophagy using GFP: MoAtg8 in both Guy11 and the Δ*Moend3* mutant. GFP was observed in 76.7% of vacuoles of Guy11, but 15.2% in Δ*Moend3* (Fig 5C and 5D).

Interestingly, the expression of the *MST7*^S212D S216E^ allele promoted GFP:MoAtg8 to enter the 68.3% of vacuoles in Δ*Moend3*. This phenomenon was further examined by the GFP:MoAtg8 proteolysis assay. Total proteins were extracted from strains expressing GFP:MoAtg8 following 0, 2 and 5 h of nitrogen starvation. The full-length GFP:MoAtg8 (41-kDa) and cleaved free GFP were detected (Fig 5E). In Guy11, the level of full-length GFP:MoAtg8 decreased as the time of nitrogen starvation increases. This was not observed in the Δ*Moend3* mutant. Meanwhile, the expression of the *MoMST7*^S212D S216E^ allele accelerated the breakdown of GFP:MoAtg8 in Δ*Moend3* (Fig 5E). Based on these results, we concluded that MoEnd3 is important for autophagy, and autophagy defect in Δ*Moend3* is possibly caused by a defect in Pmk1 phosphorylation.

### MoEnd3 function is regulated by MoArk1-mediated phosphorylation

**Fig 6:**
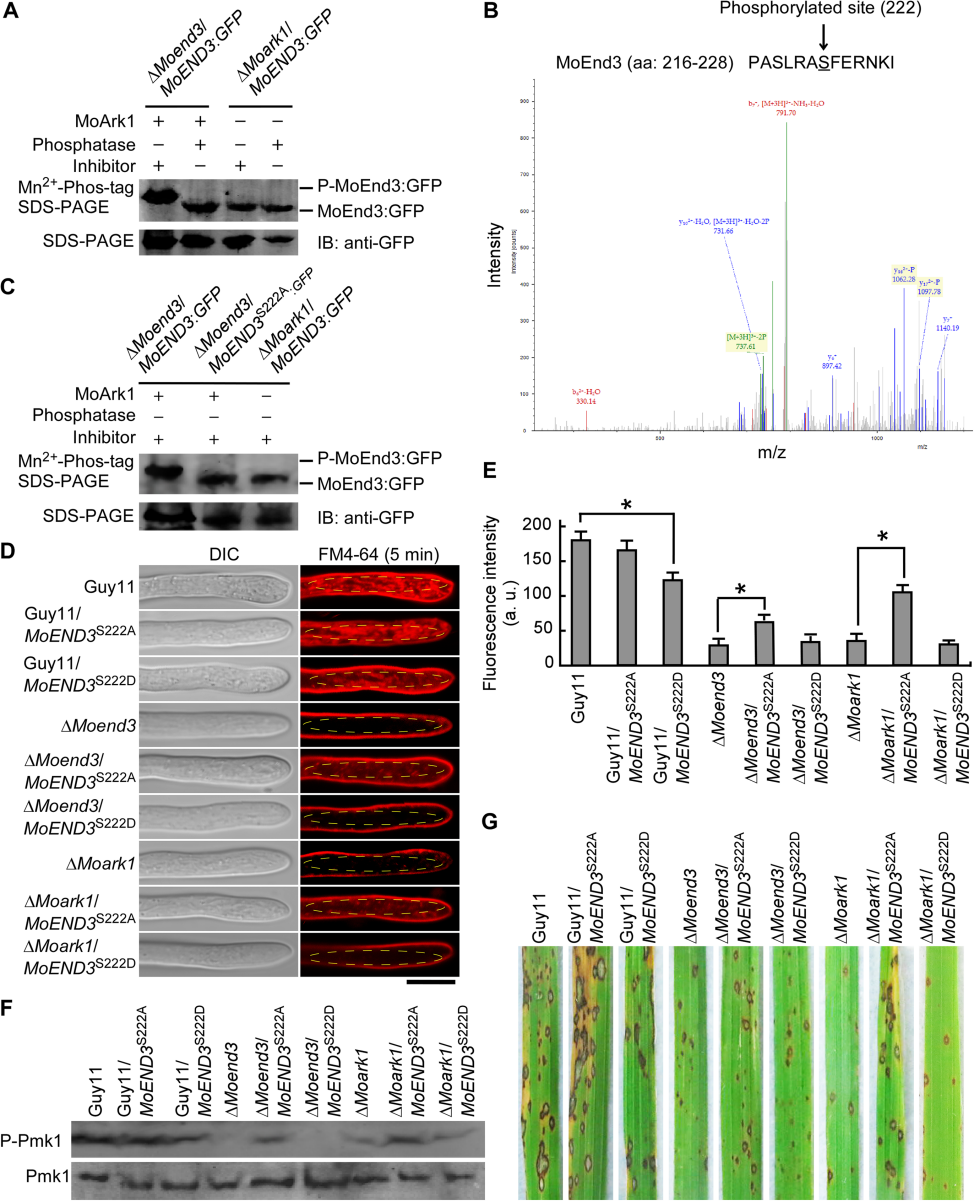
MoEnd3 phosphorylation requires MoArk1. (A) MoEnd3:GFP proteins treated with phosphatase Inhibitor or phosphatase were separated by Mn^2+^-Phos-tag SDS-PAGE and normal SDS-PAGE respectively, and were probed with GFP antibody. (B) MoEnd3 phosphopeptide (PASLRASFERNKI) in the strain expressing *MoARK1* was identified by mass spectrometer analysis and the phosphorylated site was Ser-222. (C) MoEnd3:GFP protein was extracted from the strain expressing MoArk1 and not expressing MoArk1, respectively. MoEnd3^S222A^:GFP protein was extracted from the strain expressing MoArk1. Then these proteins were separated by Mn^2+^-Phos-tag SDS-PAGE and normal SDS-PAGE, respectively, and probed with GFP antibody. (D) Hyphae were examined by fluorescence microscopy following 5 min FM4-64 staining. The selected regions where fluorescence was measured by ImageJ software were labeled by ellipse frame. Bars = 5 μm. (E) The bar chart shows mean fluorescence intensity at the hyphal tip region calculated using ImageJ software. At least 15 hyphae were measured for each strain. Asterisks represent significant differences (P < 0.01). (F) Pmk1 phosphorylation level was detected by applying phosphor-Pmk1 antibody. Endogenous Pmk1 level was detected by the Pmk1 antibody. (G) Pathogenicity assay on rice with the MoEnd3 phosphorylation site mutants. Photographs were taken following 7 days of inoculation.

Given that MoEnd3 interacts with MoArk1, a serine/threonine protein kinase, we tested whether the activity of MoEnd3 is regulated by MoArk1 through protein phosphorylation. Mn^2+^-Phos-tag SDS PAGE was thus performed to detect the phosphorylation of MoEnd3. Phosphorylated proteins in Mn^2+^-Phos-tag SDS PAGE are visualized as slower migrating bands compared with the corresponding unphosphorylated proteins [36]. We extracted the MoEnd3:GFP protein from the Δ*Moend3*/*MoEND3*:*GFP* strain. Then the protein was treated with phosphatase or phosphatase inhibitor, and was separated in Mn^2+^-Phos-tag SDS PAGE followed by analysis with the GFP antibody. The band of MoEnd3:GFP treated with the inhibitor migrated slower than that treated with phosphatase (Fig 6A), indicating that phosphorylation occurs in MoEnd3:GFP. In contrast, the band of MoEnd3:GFP from the Δ*Moark1*/*MoEND3*:*GFP* strain migrated as fast as that of the unphosphorylated MoEnd3:GFP protein treated with phosphatase (Fig 6A), indicating that MoEnd3 phosphorylation is dependent on MoArk1.

Additionally, mass spectrometry was used to identify potential phosphorylated site(s) in MoEnd3. In the strain expressing *MoARK1*, one MoEnd3 peptide containing a phosphorylated Ser-222 was detected (Fig 6B), in contrast to none found in the *MoARK1* deletion strain. We expressed the MoEnd3 Ser-222 to Ala allele linked to GFP in Δ*Moend3* and examined the phosphorylation level of MoEnd3^S222A^:GFP protein using Mn^2+^-Phos-tag SDS PAGE. The result showed that the band of MoEnd3^S222A^:GFP migrated as fast as the band of MoEnd3:GFP extracted from the Δ*Moark1*/*MoEND3*:*GFP* strain (Fig 6C), suggesting that MoEnd3^S222A^:GFP is a unphosphorylated protein and MoEnd3 Ser-222 is a specific site for MoArk1-mediated phosphorylation.

In *S*. *cerevisiae*, Ark1p/Prk1p kinases initiate phosphorylation to inhibit endocytic protein functions and promote disassembly of endocytic proteins at the late stage of endocytosis [19]. To further determine whether MoEnd3 function is regulated by MoArk1-mediated phosphorylation at Ser-222, the constructs of the constitutively unphosphorylated MoEnd3 S222A and phosphomimetic MoEnd3 S222D mutants were introduced into Δ*Moend3*, Δ*Moark1*, and Guy11, respectively. Endocytosis was observed following 5 min of hyphal exposure to FM4-64. We found that *MoEND3*^S222A^ and *MoEND3*^S222D^ expressions could not restore endocytosis to Δ*Moend3* and Δ*Moark1* (Fig 6D and 6E). However, we noticed that *MoEND3*^S222A^ expression mildly promoted endocytosis. But the *MoEND3*^S222D^ expression impaired endocytosis in Guy11, and showed no rescue effect on endocytosis in Δ*Moend3* and Δ*Moark1*, suggesting the constitutively phosphorylated MoEnd3 interferes with normal MoEnd3 function.

We further extracted proteins from appressoria or germinated conidia incubated for 8 h expressing *MoEND3*^S222A^ and *MoEND3*^S222D^ and performed Western blot analysis using the phosphor-Pmk1 antibody. We found that *MoEND3*^S222A^ expression could elevate Pmk1 phosphorylation levels to some degree in Δ*Moend3* and Δ*Moark1*, in contrast to *MoEND3*^S222D^ that was unable to induce Pmk1 phosphorylation in Δ*Moend3* (Fig 6F). In addition, the appressorium formation assay showed the Δ*Moend3*/*MoEND3*^S222A^ strain, but not the Δ*Moend3*/*MobEND3*^S222D^ strain, had a higher appressorium formation rate than Δ*Moend3* after 10 and 16 h of incubation (S6 Fig). Pathogenicity assay showed only *MoEND3*^S222A^ expression could partially rescue virulence ofΔ*Moend3* and Δ*Moark1*. Taken together, we concluded that the function of MoEnd3 is negatively regulated by MoArk1-dependent Ser-222 phosphorylation and that this regulation is important for endocytosis, Pmk1 phosphorylation, and virulence.

### MoEnd3 has a role in suppressing rice innate immunity

Plants protect themselves against pathogens by evolving multiple layers of innate immunity, which is often associated with the hypersensitive response (HR), reactive oxygen species (ROS) accumulation, and the induction of pathogenesis-related (PR) genes [37, 38]. We hypothesized that small lesions and limited IH growth by Δ*Moend3* are likely the results of the mutant being unable to suppress the host defense system. We thus measured host ROS production and HR induction using 3, 3’-diaminobenzidine (DAB) and Trypan blue staining, respectively [39–41] and found significant ROS accumulation or HR occurring at 36 hpi in over 50% of rice cells infected by Δ*Moend3*, compared with less than 20% by Guy11 and the complemented strains (S7A, S7B, S7C and S7D Fig).

Diphenyleneiodonium (DPI) functions as a flavoenzyme inhibitor that prevents the activation of NADPH oxidases necessary for ROS generation in plants [41, 42]. When treated with DPI, 51.7% of rice cells infected by Δ*Moend3* displayed improved IH grow that 36 hpi and these IH were able to spread to neighboring cells (S7E Fig), indicating that IH growth of Δ*Moend3* was arrested by strong plant defense reaction. We examined the transcript levels during the early stages of infection (0–36 hpi) of four rice pathogenesis-related (PR) genes (*PR1a*, *PAD4*, *CHT1* and *AOS2*) involved in the salicylic acid and jasmonic acid pathways [5, 42, 43] by qRT-PCR and results indicated significantly higher transcription levels of all PR genes elicited by Δ*Moend3* infection than by Guy11 infection (S7F Fig).

### MoEnd3 facilitates effector secretion

**Fig 7:**
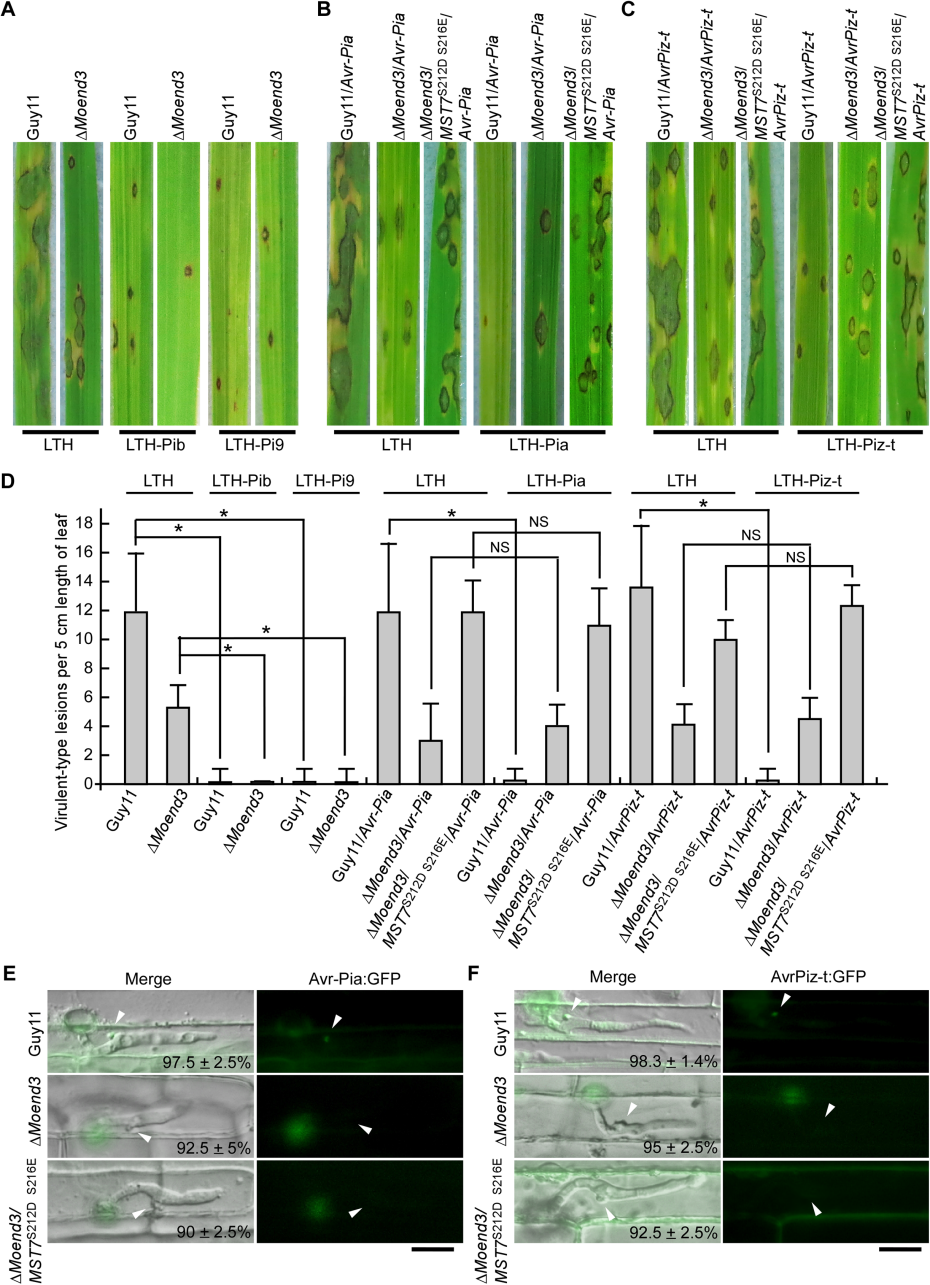
MoEnd3 is involved in secretion of effectors Avr-Pia and AvrPiz-t. (A) Pathogenicity of Guy11 and Δ*Moend3* was assayed on rice LTH, LTH-Pib and LTH-Pi9. (B) Effector *AVR*-*Pia* gene was expressed in Guy11, Δ*Moend3*, and Δ*Moend3*/*MST7*^S212DT216E^. Pathogenicity of these strains was assayed on rice LTH and LTH-Pia. (C) Effector *AVRPiz-t* gene was expressed in Guy11, Δ*Moend3*, and Δ*Moend3*/*MST7*^S212DT216E^. Pathogenicity of these strains was assayed on rice LTH and LTH-Piz-t. (D) The bar chart shows quantification of the virulent-type lesions per 5 cm length of leaf. Error bars represent SD. Asterisk represent significant difference and NS represent no significant difference. (E) Images of BICs in the rice sheath cells infected by strains expressing Avr-Pia:GFP. Merged images show DIC and GFP channel. White arrows indicate the BICs. The percentage ± SD of the types of BIC showed were recorded from three independent experiments. In each experiment, 40 BICs were observed for each strain at 24 hpi. Bar = 10 μm. (F) Images of BICs in the rice sheath cells infected by strains expressing AvrPiz-t:GFP. Merged images show DIC and GFP channel. White arrows indicate the BICs. The percentage ± SD of the types of BIC showed was recorded from three independent experiments. In each experiment, 40 BICs were observed for each strain at 24 hpi.

During the early stages of infection, *M*. *oryzae* is believed to secrete effector proteins to suppress PTI and facilitate its own growth within rice tissues. The strong immunity triggered by Δ*Moend3* led us to hypothesize that the mutant may be impaired in effector secretion. To test whether Δ*Moend3* is defective in the secretion of AvrPib and AvrPi9 effectors, conidial suspensions were sprayed onto rice LTH (a universally susceptible rice variety), LTH-Pib (LTH harboring resistant gene *Pib*), and LTH-Pi9 (LTH harboring resistant gene *Pi9*). Guy11 produced many typical virulent-type lesions on LTH and tiny dark-brown HR-type lesions (a highly resistant response) in LTH-Pib and LTH-Pi9 (Fig 7A and 7D). The virulent-type lesions are larger than 1 mm in diameter and are considered virulent because conidia will be produced from this type of lesions under high humidity condition [44]. In contrast, the HR-type lesions are smaller than 1 mm, cannot produce conidia, and considered avirulent. Δ*Moend3* still could produce virulent-type lesions in LTH, but the lesions were much less than those produced by Guy11, and Δ*Moend3* induced the resistant response in LTH-Pib and LTH-Pi9, similar to Guy 11 (Fig 7A and 7D). These results suggested that MoEnd3 is dispensable for AvrPib and AvrPi9 triggered host immunity.

To test other effectors that are not contained in Guy11, such as Avr-Pia and AvrPiz-t, constructs containing genes encoding Avr-Pia and AvrPiz-t were introduced into Guy11 and Δ*Moend3*. Conidial suspensions of Guy11/*Avr*-*Pia* and Δ*Moend3*/*Avr*-*Pia* were sprayed onto LTH and LTH-Pia (LTH harboring resistant gene *Pia*). Guy11/*Avr*-*Pia* was found to have normal infection in LTH and induce aresistant response in LTH-Pia. However, Δ*Moend3*/*Avr*-*Pia* produced typical lesions in LTH-Pia and LTH, suggesting Avr-Pia secretion may be affected in Δ*Moend3* (Fig 7B and 7D). Similarly, Δ*Moend3*/*AvrPiz-t* was unable to cause a strong resistant response in LTH-Piz-t in comparison to Guy11/*AvrPiz-t* (Fig 7C and 7D), suggesting that *MoEND3* deletion also inhibits AvrPiz-t function.

Avr-Pia and AvrPiz-t are cytoplasmic effectors that are preferentially accumulated in the biotrophic interfacial complex (BIC) and translocated to the rice cell cytoplasm [45]. We fused Avr-Pia and AvrPiz-t with GFP, expressed them in Guy11 and Δ*Moend3*, and observed their localizations at the early stage of infection. In the cells infected by Guy11, Avr-Pia:GFP and AvrPiz-t:GFP accumulated in over 95% of BIC structures adjacent to primary hyphae (Fig 7E and 7F), in contrast to the cells infected by Δ*Moend3* in which less than 10% of BICs contained Avr-Pia:GFP and AvrPiz-t:GFP (Fig 7E and 7F).

To further demonstrate the requirement of MoEnd3 for secretion of Avr-Pia and AvrPiz-t, but not AvrPib and AvrPi9, we observed effector secretion with the strains co-expressing Avr-Pia:GFP and AvrPiz-t:GFP with AvrPib:RFP or AvrPi9:RFP. For Guy11, we found about 95% of BICs containing AvrPib:RFP or AvrPi9:RFP appeared with Avr-Pia:GFP and AvrPiz-t:GFP (S8A, S8B, S8C and S8D Fig). For Δ*Moend3*, more than 90% of BICs showed the presence of AvrPib:RFP or AvrPi9:RFP, but less than 10% of BICs with AvrPib:RFP or AvrPi9:RFP containing Avr-Pia:GFP and AvrPiz-t:GFP. Moreover, RT-PCR analysis for Avr-Pia and AvrPiz-t during infection showed that *MoEND3* deletion did not inhibit their expression (S9 Fig), which ruled out the possibility that this secretion defect of Δ*Moend3* was caused by the inhibition of effector gene expression.

**Fig 8:**
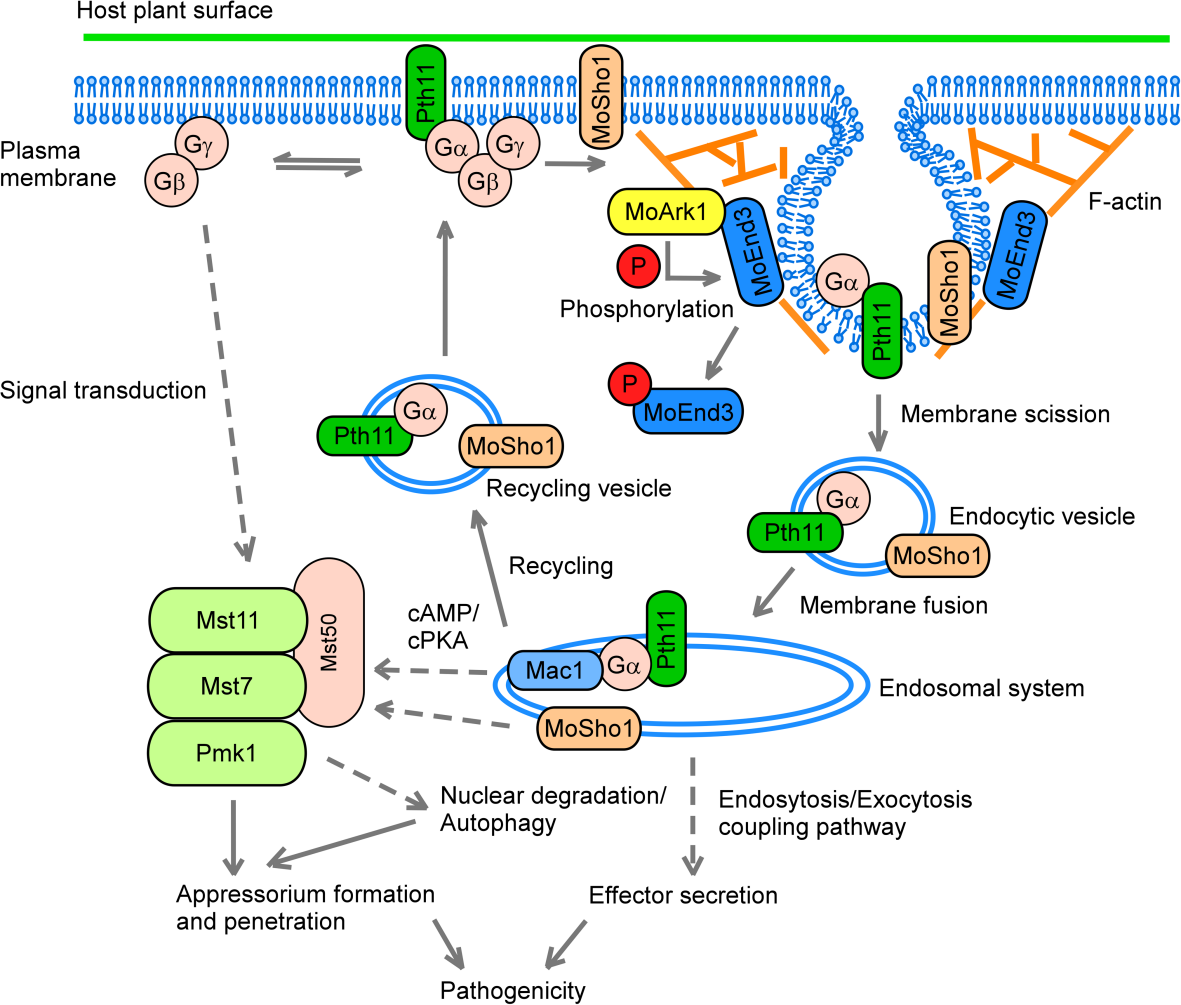
A proposed model for MoEnd3 function. During germ tube development, GPCR Pth11 and membrane sensor MoSho1 are regulated by MoEnd3-mediated endocytosis. MoEnd3 function in endocytosis is negatively regulated by MoArk1-initiated phosphorylation, leading to an efficient endocytosis. Following transport to endosomal systems by endocytic vesicles, Pth11 and MoSho1 can trigger a downstream MAPK cascade, consisting of Mst11, Mst7, Pmk1 and adaptor Mst50. The MAPK cascade facilitates successful nuclear degradation/autophagy, appressorium formation, penetration and pathogenicity. In addition, MoEnd3 is also involved in an endocytosis/exocytosis coupling pathway to facilitate effector secretion and biotrophic growth.

Interestingly, the expression of the *MST7*^S212D S216E^ allele in Δ*Moend3* was unable to induce a resistant response in rice harboring resistant genes (Fig 7B, 7C and 7D) and to enrich Avr-Pia:GFP and AvrPiz-t:GFP in BICs (Fig 7E and 7F), suggesting that the two effector secretion may be not directly regulated by Pmk1-MAPK. Moreover, the DAB staining assay indicated that Δ*Moend3*/*MST7*^S212D S216E^ failed to suppress ROS responses as effectively as Guy11 (S10 Fig), implying that the expression of the *MST7*^S212D S216E^ allele still cannot restore effector secretion required for suppressing rice innate immunity. Taken together, we concluded that MoEnd3 facilitates secretion of effectors such as Avr-Pia and AvrPiz-t, but not Avr-Pib and Avr-Pi9, though a pathway independent of Pmk1 phosphorylation.

## Discussion

Endocytosis is employed by eukaryotic cells to constitutively internalize plasma membrane-associated proteins, lipids, and other molecules for regulating many key cellular functions. In *M*. *oryzae*, this process is closely linked to fungal physiology and pathogenicity [23, 24, 45–47]. Our current studies provide evidence further supporting this conclusion. Our results show that in addition to having an important role in mating and virulence, MoEnd3-mediated endocytosis is also important for transport of the GPCR Pth11 and the membrane sensor MoSho1. Significantly, *MoEND3* deletion delayed endocytosis of Pth11 and MoSho1, resulting in delayed appressorium development. Similar to phenotypes in the strains lacking *cPKA* [48], the appressoria produced by Δ*Moend3* strains showed impaired turgor pressure, inefficient mobilization of glycogen and lipids, and a defect in host penetration. Additionally, we found that MoEnd3 function affects the Pmk1 MAPK signaling pathway. Collectively, our findings support that endocytosis is required for receptor-mediated signaling, development and pathogenesis in *M*. *orzae*.

Our findings are consistent with observations in other model organisms. For example, in mammalian cells, activation of plasma membrane receptors including receptor tyrosine kinases and GPCR by external agonists is followed by the endocytic receptor transport to the endosome. In the endosome the internalized receptors can interact with key components of various signaling pathways to activate specific signal transduction pathways [49, 50]. Furthermore, in the biotrophic plant pathogen *Ustilago maydis*, studies of tSNARE Yup1 revealed that endocytosis controls GPCR Pra1-mediated signaling. Yup1 is co-localized with Rab5-marked early endosomes. A temperature-sensitive mutation of *yup1* blocked the fusion of endocytic vesicles with early endosomes and the endocytic recycling pathway [51]. These defects result in depletion of the pheromone receptor Pra1 from the plasma membrane and disruption in pheromone-mediated signal transmission to downstream effectors that would normally trigger pathogenic development [51].

Autophagic cell death in the conidium is necessary for appressorium formation and infection. Previous studies have shown that a Δ*pmk1* mutant is blocked in autophagic nuclear degradation [33]. We found that constitutively activated Mst7 could accelerate autophagy in the Δ*Moend3* mutant. This supports the hypothesis that the severely delayed nuclear degradation and autophagy in Δ*Moend3* was caused by a defect in Pmk1-MAPK signaling. This is in agreement with several other studies that also found that MAPK signaling is involved in the autophagic process. In mammalian cells, members of the MAPK family including MAPK1/ERK2, MAPK8/JNK, MAPK14/p38a and MAPK15 are involved in the control of autophagy [52–54]. In *S*. *cerevisiae*, the Slt2-MAPK and Hog1-MAPK signaling pathways were found to be required for mitophagy and pexophagy [55].

Additionally, mammalian and yeast Ark1p/Prk1p serine/threonine kinases initiate phosphorylation of endocytic and actin cytoskeleton components to control endocytosis [19]. We previously reported that MoArk1 regulates endocytosis and pathogenicity and is localized to actin patches in *M*. *oryzae* [23]. Here, we demonstrated that MoEnd3 function is regulated by MoArk1 through protein phosphorylation. We further found that neither of the constitutively phosphorylated nor unphosphorylated form of MoEnd3 could properly function in endocytosis, Pmk1 phosphorylation or virulence. Strikingly, the unphosphorylated MoEnd3 could still function to partially suppress the defects of Δ*Moend3* and Δ*Moark1*. This is in contrast to the constitutively phosphorylated MoEnd3, which was completely inactive.

*M*. *oryzae* secretes effectors, such as Slp1, into rice cells to suppress host immunity [56]. IH growth of Δ*Moend3* was found to be arrested suggesting that it was inhibited by a robust host immune response. This could be due to Δ*Moend3* being unable to secrete effector molecules. Indeed, we found that the secretion of Avr-Pia and AvrPiz-t was impaired in Δ*Moend3*. This finding is in accordance with our earlier studies in which we found that Qc-SNARE MoSyn8 is required for Avr-Pia and AvrPiz-t secretion [45]. However, the secretion of AvrPib and AvrPi9 was not affected in Δ*Moend3*, suggesting that secretion of these effectors may involve mechanisms independent of MoEnd3. Moreover, when Pmk1 phosphorylation was activated by expressing the *MST7*^S212D T216E^ allele in Δ*Moend3*, the secretion of Avr-Pia and AvrPiz-t was still impaired, suggesting that these mechanisms are also independent of Pmk1 signaling. It would be interesting to identify such mechanisms in future studies.

Previous studies indicated that there are two distinct effector secretion systems functioning in *M*. *oryzae* [2]. The cytoplasmic effectors such as Pwl2 are preferentially accumulated in BIC, and their secretion depends on the t-SNARE protein MoSso1 and exocyst components MoExo70 and MoSec5. The secretion of apoplastic effectors, such as Bas4, follows the Golgi-dependent secretion pathway [2]. Some studies also indicated that endocytosis and exocytosis/secretion are obligatorily coupled [57, 58]. In *S*. *cerevisiae*, the perturbation of She4p affects endocytosis and defects in endocytosis result in a slow motion of exocytic vesicles during polarity establishment [59]. This decreased exocytosis could reflect in defects in endocytic recycling of components required for membrane fusion, including certain SNARE proteins [59]. Therefore, it is likely that MoEnd3-mediated endocytosis affects secretion of certain effector proteins and that delayed endocytosis in Δ*Moend3* could also affect movement of certain exocytic vesicles required for transporting effector proteins. Ultimately, inhibition of effector secretion could attenuate *M*. *orzae* pathogenicity.

In summary, our studies demonstrate that the endocytic protein MoEnd3 is required for blast fungus growth and development, endocytic transport of pathogenic GPCRs, interaction with the rice host, and pathogenicity. Together with MoArk1, MoEnd3 exhibits a regulatory function for multiple processes, including appressorium development and function, autophagy, Pmk1 MAPK transduction, and signaling and regeneration of Pth11 and MoSho1 (Fig 8). Given that endocytosis is closely coupled with exocytosis, MoEnd3 could have additional roles in facilitating effector secretion to suppress host defenses.

## Methods

### Strains and culture conditions

*M*. *oryzae* Guy11 was used as the parental wild type strain in this study. All strains were cultured on complete medium (CM) agar plates. Liquid CM medium was used to prepare the mycelia for DNA and RNA extraction. For conidia production, strains were maintained on straw decoction and corn (SDC) agar media at 28°C for 7 days in the dark followed by 3 days of continuous illumination under fluorescent light [42].

### Mating

Plugs of mutant and the wild type strain Guy11 (MAT1-2) and the mating partner strain TH3 (MAT1-1) were point inoculated 3 cm apart on oatmeal agar medium and incubated at 20°C under constant fluorescent light for 3 to 4 weeks [60].

### Targeted *MoEND3* deletion and the Δ*Moend3* mutant complementation

The *MoEND3* deletion mutant was generated using the standard one-step gene replacement strategy [61]. First, two approximate 1.0 kb of sequences flanking of *MoEND3* (MGG_06180) were amplified with two primer pairs *MoEND3*-F1/*MoEND3*-R1, *MoEND3*-F2/*MoEND3*-R2, the products of *MoEND3* were digested with restriction endonucleases (*Eco*RI and *Sal*I, *Spe*I and *Sac*II) and ligated with the *HPH* cassette released from pCX62. The protoplasts of wild type Guy11 were transformed with the vectors for targeted gene deletion by inserting the hygromycin resistance *HPH* marker gene cassette into the two flanking sequences of the *MoEND3* gene. For selecting hygromycin-resistant transformants, CM plates were supplemented with 250 μg/ml hygromycin B (Roche, USA).

To generate complementary construct pYF11-*MoEND3*, the gene sequence containing the *MoEND3* gene and 1.0 kb native promoter was amplified with *MoEND3*-comF/*MoEND3*-comR. Yeast strain XK1-25 was co-transformed with this sequence and *Xho*I-digested pYF11 plasmid. Then the resulting yeast plasmid was expressed in *E*. *coli*. To generate the complementary strain, the pYF11-*MoEND3* construct containing the bleomycin-resistant gene for *M*. *oryzae* transformants screen was introduced into the Δ*Moend3* mutant [61].

### Southern blot analysis

*Eco*RV was used to digest the genomic DNA from wild-type strain Guy11 and the Δ*Moend3* mutant. The digest products were separated in 0.8% agar gel and were hybridized with the *MoEND3* gene probe. The probe was designed according to the disruption strategy and was amplified from Guy11 genomic DNA using primers *MoEND3*-InterF/*MoEND3*-InterR. To confirm *MoEND3* replacements, labeled *MoEND3* probe was used to hybridize the *Eco*RV-digested genomic DNA from the Δ*Moend3* mutant and wild-type Guy11. The copy number of *HPH* gene in the Δ*Moend3* mutant was detected using labeled *HPH* fragments that amplified from the plasmid of pCB1003 with primers FL1111/FL1112. The whole hybridization was carried out according to the manufacturer’s instruction for DIG-High Prime [61].

### Pathogenicity assay

Conidia were harvested from 10-day-old SDC agar cultures, filtered through three layers of lens paper and re-suspended to a concentration of 5×10^4^ spores/ml in a 0.2% (w/v) gelatin solution. Two-week-old seedlings of rice (cv. CO39) and 7-day-old seedlings of barley (*Hordeum vulgare* cv. Four-arris) were used for pathogenicity assays. For spray inoculation, 5 ml of a conidial suspension of each treatment were sprayed onto rice with a sprayer. Inoculated plants were kept in a growth chamber at 28°C with 90% humidity and in the dark for the first 24 h, followed by a 12 h/12 h light/dark cycle. Lesion formation in rice and barley was observed after 7 and 5 days, respectively [60].

### Rice sheath and barley epidermis penetration assays, appressorium formation assay, appressorium turgor determination and glycogen/lipid staining

For infection assay with rice tissues, conidia were re-suspended to a concentration of 1×10^5^ spores/ml in a 0.2% (w/v) gelatin solution. 3-week-old rice cultivar CO-39 was inoculated with 100 μl of conidial suspension on the inner leaf sheath cuticle cells and incubation under humid conditions at 28°C. The leaf sheaths were observed under Zeiss Axio Observer A1 inverted microscope at 36 hpi. For barley epidermis penetration assays, conidia were suspended to a concentration a concentration of 5×10^4^ spores/ml in a 0.2% (w/v) gelatin solution. Droplets (10 μl) of conidial suspension were placed on detached barley leaf epidermis. The barley epidermis was observed under Zeiss Axio Observer A1 inverted microscope at 24 hpi.

Conidia were harvested from 10-day-old cultures, filtered through three layers of lens paper, and re-suspended to a concentration of 5×10^4^ spores/ml in sterile water. For appressorium formation assay, droplets (30 μl) of conidial suspension were placed on plastic cover slips (Fisher Scientific, St Louis, MO, USA) under humid conditions at 28°C [62]. Appressorium turgor was determined by cell collapse assay using a 1–4 molar concentration of glycerol solution. The percentages of conidia germinating and conidia forming appressoria were determined by microscopic examination of at least 100 conidia. To visualize glycogen and lipid, KI solution and Neil red were used as described [48]. All the samples were observed under Zeiss Axio Observer A1 inverted microscope (40×).

### DAB and Trypan blue staining, and the penetration assay with DPI treatment

For DAB staining assay, rice tissues infected by strains at 36 hpi were stained with 1 mg/ml DAB (Sigma-Aldrich) solution (pH 3.8) for 8 h and destained with an ethanol/acetic acid solution (ethanol/acetic acid = 98:2, v/v) for 1 h. For Trypan blue staining assay, rice tissues infected by strains at 36 hpi were stained with a 2.5 mg/ml Trypan blue solution for 1 h and destained in 2.5 g/ml lactophenol for 1 h. For evaluating the growth of IH in ROS-suppressed rice sheath, a conidial suspension (1×10^5^ spores/ml) treated with 0.5 μm DPI was inoculated into the rice sheath for 36 h. All the samples were observed under Zeiss Axio Observer A1 inverted microscope (40×).

### RT-PCR analysis

For detection of the rice PR gene transcription during infection stage, total RNA samples were extracted from plants inoculated with the wild-type strain or mutant at 0, 24, 48, and 72 hpi. Transcription of elongation factor 1a gene (Os03g08020) was used as endogenous control in *O*. *sativa*. For detection of *AVR*-*Pia* and *AVRPiz*-*t* transcription during infection stage, total RNA samples were extracted from the strains at 24 and 48 hpi. Transcription of actin gene (XP 003719871.1) was used as endogenous control. The qRT-PCR was run on the Applied Biosystems 7500 Real Time PCR System with SYBR *Premix Ex Taq* (Perfect Real Time, Takara, Japan). Normalization and comparison of mean Ct values were performed as previously described [42].

### Yeast two-hybrid assay

Bait constructs were generated by cloning *MoARK1* and *MoACT1* full-length cDNAs into pGBKT7, respectively. *MoEND3* full-length cDNA was cloned into pGADT7 as the prey construct. The prey and bait constructs were confirmed by sequencing analysis. The yeast strain AH109 was transformed with the bait and prey constructs as the description of BD library construction & screening kit (Clontech, USA). The Trp^+^ and Leu^+^ transformants were isolated and assayed for growth on SD-Trp-Leu-His-Ade medium [63].

### BiFC assay for MoEnd3-MoArk1 interaction

The *MoEND3*-YFP^N^ plasmid was generated by cloning the *MoEND3* gene with a native promoter into the vector pHZ65 containing hygromycin-resistant gene. The *MoARK1* gene with a native promoter was cloned into the vector pHZ68 containing bleomycin-resistant gene to generate the *MoEND3*-YFP^C^ plasmid. The two plasmids were introduced into protoplasts of wild type Guy11. Transformants resistant to both hygromycin and bleomycin were isolated and examined using fluorescence microscopy (Zeiss Axio Observer A1 inverted microscope, 40×).

### *In vitro* protein binding assays

To construct the plasmids of *GST*-*MoEND3*, *His*-*MoARK1* and His-*ACT1*, full-length cDNA of *MoEND3* was amplified and inserted into the vector pGEX4T-2, and full-length cDNAs of *MoARK1* and *MoACT1* were amplified and inserted into the vector pET-32a, respectively. Then these plasmids were expressed in *E*. *coli* strain BL21 (DE3) and bacterial cells were collected and treated by lysis buffer (10 mM Tris-HCl [pH 7.5], 150 mM NaCl, 0.5 mM EDTA, 0.5% Triton x-100). To confirm expression of the GST or His fusion proteins, bacterial lysates were separated by SDS-PAGE gel followed by Coomassie blue staining. In the binding assay for His-MoArk1 and GST-MoEnd3, bacterial lysate containing His-Ark1 protein was incubated with 30 μl Ni-NTA agarose beads (Invitrogen, Shanghai, China) for 1 h at 4°C. Then the beads were washed for five times, incubated with bacterial lysate containing GST-MoEnd3 for 1 h at 4°C, washed for five times again and boiled for elution. The elution was probed with His and GST antibodies (Abmart, Shanghai, China). In the binding assay for His-MoAct1 and GST-MoEnd3, bacterial lysate containing GST-MoEnd3 protein was incubated with 30 μl GST agarose beads (Invitrogen, Shanghai, China) for 1 h at 4°C. Then the beads were washed for five times, incubated with bacterial lysate containing His-MoAct1 for 1 h at 4°C and boiled for elution. The elution was probed with His and GST antibodies (Abmart, Shanghai, China).

### Plasmid construction

To construct plasmids of *MoARK1*:*FLAG*, *PTH11*:*GFP*, *MoMSB2*:*GFP*, *MoSHO1*:*GFP*, *MST7*^S212D T216E^ (RP27 promoter), *MoEND3*:*GFP*, *MoEND3*^S222A^:*GFP*, *MoEND3*^S222D^:*GFP*, *Lifeact*:*RFP* (RP27 promoter), *H1*:*RFP*, *Avr*-*Pia*:*GFP*, *AvrPiz*-*t*:*GFP*, *AvrPi9*:*RFP* and *AvrPib*:*RFP*, their gene fragments were amplified with primers listed in S3 Table and inserted into pYF11 plasmid by transformation with yeast XK1-25 strain. Yeast transformants were isolated from the SD-Trp plates and resulting constructs were amplified by expression in *E*. *coli*.

### Assays with FM4-64, actin inhibitor Latrunculin B, cycloheximide and anti-microtubule drug benomyl

FM4-64 (Molecular Probes Inc., Eugene, OR, USA) was solved in distilled water to a final concentration 5 μg/ml. For assaying with hyphae, strains were grown on CM liquid medium for 16 h at 28°C. Before observation, hyphae were washed with distilled water and strained with FM4-64 on glass slide. For assaying with germinated conidia, conidia were inoculated on the coverslips with hydrophobic surface. After 3 h, the dye was added to the conidia for 10 min. Then samples were washed with distilled water. Latrunculin B (LatB) (Cayman, USA) is stocked in DMSO in a concentration of 25 mg/ml. Conidia incubated on the coverslips with hydrophobic surface were treated with LatB (final concentration 0.1 μg/ml) for 30 min, while the controls were treated with 5% DMSO. Then samples were washed with distilled water. Cycloheximide (MedChemExpress, USA) was solved in distilled water and the germinated conidia were treated with a final concentration 10 μg/ml for 10 min. Then samples were washed with distilled water. Benomyl (Aladdin, Shanghai, China) was solved in 0.1% DMSO and added to germinated conidia with a final concentration 1 μg/ml. Then the samples were washed with distilled water. All the samples were observed under a fluorescence microscope (Zeiss LSM710, 63× oil). The filter cube sets: GFP (excitation spectra: 488 ± 10 nm, emission spectra: 510 ± 10 nm), FM4-64 (excitation spectra: 535 ± 20 nm, emission spectra: 610 ± 30 nm). Exposure time: 800 ms.

### Imaging of effector secretion

The conidial suspensions (1×10^5^ conidia/ml in a 0.2% gelatin) were injected into rice sheath from 3-week-old rice seedlings (cv. CO39). The BICs in the infected rice cells were observed using fluorescence microscopy (Zeiss Axio Observer A1 inverted microscope, 40×) at 24 hpi and the images were captured immediately. The filter cube sets: GFP (excitation spectra: 488 ±10 nm, emission spectra: 510 ± 10 nm), RFP (excitation spectra: 561 ± 10 nm, emission spectra: 610 ± 10 nm). Exposure time: 800 ms.

### Western blotting for Pmk1 detection

About 150 to 200 mg of mycelia were ground into powder in liquid nitrogen and resuspended in 1 ml of extraction buffer (10 mM Tris-HCl [pH 7.5], 150 mM NaCl, 0.5 mM EDTA, 0.5% Triton x-100) with fresh added 1 mM PMSF and 10 μl of protease inhibitor cocktail (Sigma, Shanghai, China). Total proteins were separated on a 12% SDS-PAGE gel and transferred to nitrocellulose membranes. The p44/42 MAPK (Erk1/2) antibody (Cell Signaling Technology, USA) was used to detect endogenous Pmk1 expression. The phospho-p44/42 MAPK (Erk1/2) (Thr202/Tyr204) antibody (Cell Signaling Technology, USA) was used to detect phophorylated Pmk1.

### FRAP assay

Thegerminated conidia with 3 h of incubation were treated with cycloheximide and benomyl as described. FRAP were performed using a fluorescence microscope Zeiss LSM710. Regions containing Pth11:GFP and MoSho1:GFP in germ tube were selected for photo-bleaching. The photobleaching was carried out using an Argon-multiline laser at a wavelength of 488 nm with 90% laser power and 150 iterations in ROI. Images were acquired with 2% laser power at a wavelength of 488 nm every 5 sec. For quantitative analyses, fluorescence intensity was measured using the ZEISS ZEN blue software and fluorescence recovery curves were fitted using following formula: F(t) = F_min_ + (F_max_ − F_min_)(1-exp^−kt^), where F(t) is the intensity of fluorescence at time t, F_min_ is the intensity of fluorescence immediately post-bleaching, F_max_ is the intensity of fluorescence following complete recovery, and k is the rate constant of the exponential recovery [64]. Mobile Fraction was calculated as the following formula: Mf = (F_end_ − F_0_)/(F_pre_ − F_0_), where F_end_ is the stable fluorescent intensity of the punctae after sufficient recovery, F_0_ is the fluorescent intensity immediately after bleaching, and F_pre_ is the fluorescent intensity before bleaching [65].

### Phosphorylation analysis with phos-tag gel

The *MoEND3*:*GFP* fusion construct was introduced into Δ*Moend3* and Δ*Moark1* mutants, respectively. The proteins extracted from mycelium were resolved on 8% SDS-polyacrylamide gels prepared with 50 μM acrylamide-dependent Phos-tag ligand and 100 μM MnCl_2_ as described [36]. Gel electrophoresis was run at 80 V for 3–6 h. Prior to transfer, gels were equilibrated in transfer buffer containing 5 mM EDTA for 20 min two times and then in transfer buffer without EDTA for 10 min. Protein transfer from the Mn^2+^-phos-tag acrylamide gel to the PVDF membrane was performed overnight at 80 V at 4°C, and then the membrane was analyzed by Western blotting using the anti-GFP antibody.

### Mass spectrometric analysis

To identify phosphorylation sites of targeted proteins, samples were separated on 10% SDS PAGE. The gel bands corresponding to the targeted protein were excised from the gel, reduced with 10 mM of DTT and alkylated with 55 mM iodoacetamide. In gel digestion was carried out with the trypsin/lys-c mix (Promega, USA) in 50 mM ammonium bicarbonate at 37°C overnight. The peptides were extracted using ultrasonic processing with 50% acetonitrile aqueous solution for 5 min and with 100% acetonitrile for 5 min. The extractions were then centrifuged in a speed to reduce the volume. A liquid chromatography–mass spectrometry (LC–MS) system consisting of a Dionex Ultimate 3000 nano-LC system (nano UHPLC, Sunnyvale, CA, USA), connected to a linear quadrupole ion trap Orbitrap (LTQ Orbitrap XL) mass spectrometer (ThermoElectron, Bremen, Germany), and equipped with a nanoelectrospray ion source was used for our analysis. For LC separation, an Acclaim PepMap 100 column (C18.3 μm, 100 Å) (Dionex, Sunnyvale, CA, USA) capillary with a 15 cm bed length was used with a flow rate of 300 nL/min. Two solvents, A (0.1% formic acid) and B (aqueous 90% acetonitrile in 0.1% formic acid), were used to elute the peptides from the nanocolumn. The gradient went from 5% to 40% B in 80 min and from 40% to 95% B in 5 min, with a total run time of 120 min. The mass spectrometer was operated in the data-dependent mode so as to automatically switch between Orbitrap-MS and LTQ-MS/MS acquisition. Survey full scan MS spectra (from m/z 350 to 1800) were acquired in the Orbitrap with a resolution r = 60,000 at m/z 400, allowing the sequential isolation of the top ten ions, depending on signal intensity. The fragmentation on the linear ion trap used collision-induced dissociation at a collision energy of 35 V. Protein identification and database construction were processed using Proteome Discoverer software (1.2 version, Thermo Fisher Scientific, Waltham, MA, USA) with the SEQUEST model. MS/MS-based peptide identifications were accepted if they could be established at greater than 95.0% probability, as specified by the Peptide prophet algorithm.

### Accession numbers

Gene sequences can be found in the GenBank database under the following accession numbers: *MoEND3* (MGG_06180), *MoARK1* (MGG_11326), *MoACT1* (MGG_03982), *PTH11* (MGG_05871), *MoMSB2* (MGG_06033), *MoSHO1* (MGG_09125) and *MST7* (MGG_00800).

## Supporting information

S1 Fig Targeted *MoEND3* deletion was confirmed by Southern blot analysis. Southern blot analysis of the *MoEND3* gene deletion mutants with gene specific probe (probe1) and hygromycin phosphotransferase (HPH) probe (probe2). Thick arrows indicate orientations of the *MoEND3* and *HPH* genes. Thin lines below the arrows indicate sequence-specific gene probes.
(TIF)

S2 Fig MoEnd3 is involved in mating. Perithecia production was photographed following three weeks of incubation. Cross between TH3 (MAT1-1) and Guy11 (MAT1-2) represents the positive control. Cross between the Δ*Moend3* mutant and TH3 failed to produce peritheria or asci. Cross between the complemented strain and TH3 produced normal peritheria and asci. Bars = 20 μm.
(TIF)

S3 Fig MoEnd3 contributes to glycogen and lipid translocation and degradation. (A) Conidia were incubated on hydrophobic surface. Samples were stained with iodine solution at different time points and yellowish-brown glycogen deposits became visible immediately. Bars = 10 μm. (B) The percentage of conidia containing glycogen was recorded with observing at least 100 germinated conidia for each sample. The experiment was repeated three times. Error bars represent SD and asterisks represent significant differences (P < 0.01). (C) Conidia were allowed to germinate on hydrophobic surface. Samples were stained for the presence of lipid bodies by using Nile red. Bars = 10 μm. (D) The percentage of conidia containing abundant lipids was recorded with observing at least 100 germinated conidia. The experiment was repeated three times. The error bars represent SD and asterisks represent significant differences.
(TIF)

S4 Fig Penetration assay with barley leaves. Detached barley leaves from 7-day old barley seedlings were inoculated with conidial suspension. IH on barley epidermal cells was observed at 24 hpi and 4 types of IH were quantified and statistically analyzed. Micrographs show 4 types of IH in barley epidermal cells.
(TIF)

S5 Fig Co-localization of FM4-64 with GFP:Rab5, GFP:Rab7 and CMAC in germ tube. (A) Most of FM4-64 in germ tube was located to GFP:Rab5 labeled structures (early endosomes) which were distinct from CMAC stained vacuoles. (B) Co-localization of FM4-64 with GFP:Rab7 known to mark late endosomes was rarely occurred in germ tube. CMAC stained vacuoles did not appear in germ tube.
(TIF)

S6 Fig MoEnd3 Ser-222 phosphorylation is important for appressorium formation. Images were taken from the strains after 10 and 16 h of incubation on hydrophobic surfaces. Bars = 10 μm. Appressorium formation rates were calculated and statistically analyzed. Error bars represent SD and asterisks represent significant differences.
(TIF)

S7 Fig MoEnd3 is involved in suppressing rice defense system. (A) Infected rice tissues were stained with DAB. DAB staining indicates that ROS accumulated in the rice cells infected by the Δ*Moend3* mutant but not by Guy11 and the complemented strain at 36 hpi. Bars = 10 μm. (B) The percentage of infected rice cells stained with DAB (n = 50). Error bars represent SD and asterisk represents significant difference (P < 0.01). (C) Infected rice tissue was stained with Trypan blue. HR occurs in rice cells infected by the Δ*Moend3* mutant but not Guy11 and the complemented strain. Bars = 10μm. (D) The percentage of the rice cells stained with Trypan blue (n = 50). Error bars represent SD and asterisk represents significant difference (P < 0.01). (E) IH growth in rice cells treated with DPI. When rice tissue was treated with 0.5 mM DPI dissolved in DMSO, the Δ*Moend3* mutant partly restored growth in rice cells and extended IH to neighboring rice cells. The samples treated with DMSO and without DPI were used as negative controls. The percentage ± SD of the patterns showed was given. Bars = 10μm. (F) Expressions of rice pathogenesis-related genes (*PR1a*, *AOS2*, *CHT1* and *PAD4*) were analyzed by qRT-PCR during early infection stage. RNA samples were collected from rice plants infected by Guy11 and Δ*Moend3* mutant at 0, 8, 16, 24, and 36 hpi. Error bars represent the standard deviation and asterisks represent significant differences (P < 0.01).
(TIF)

S8 Fig MoEnd3 facilitates secretion of Avr-Pia and AvrPiz-t but not AvrPib and AvrPi9. (A) Images of BICs in the rice sheath cells infected by strains expressing Avr-Pia:GFP and AvrPib:RFP. Merged images show GFP and RFP channels. White arrows indicate the BICs. The percentage ± SD (standard deviation) of the types of BIC showed was recorded from three independent experiments. In each experiment, 20 BICs containing AvrPib:RFP were observed for each strain at 24 hpi. Bar = 10 μm. (B) Images of BICs in the rice sheath cells infected by strains co-expressing AvrPiz-t:GFP and AvrPib:RFP. The percentage ± SD of the types of BIC showed was recorded from three independent experiments. In each experiment, 20 BICs containing AvrPib:RFP were observed for each strain at 24 hpi. Bar = 10 μm. (C) Images of BICs in the rice sheath cells infected by strains co-expressing Avr-Pia:GFP and AvrPi9:RFP. The percentage ± SD of the types of BIC showed was recorded from three independent experiments. In each experiment, 20 BICs containing AvrPi9:RFP were observed for each strain at 24 hpi. Bar = 10 μm. (D) Images of BICs in the rice sheath cells infected by strains co-expressing AvrPiz-t:GFP and AvrPi9:RFP. The percentage ± SD of the types of BIC showed was recorded from three independent experiments. In each experiment, 20 BICs containing AvrPi9:RFP were observed for each strain at 24 hpi. Bar = 10 μm.
(TIF)

S9 Fig Transcription of Avr-Pia and AvrPiz-t is not inhibited in Δ*Moend3* during Infection. RNA samples were collected from Guy11/*AVR-Pia* and Δ*Moend3*/*AVRPiz-t* at 24 and 48 hpi and the transcription level of *AVR-Pia* and *AVRPiz-t* was analyzed by RT-PCR.
(TIF)

S10 Fig Expressing *MST7*^S212D T216E^ allele partially promotes Δ*Moend3* to suppress ROS. (A) DAB was used to stain ROS in the rice sheath tissue infected by Guy11, Δ*Moend3*, Δ*Moend3*/*MST7*^S212D T216E^, and Δ*Moend3*/*MoEND3*. (B) The percentage of the infected rice cells with ROS accumulation. 50 infected cells were observed for each strain and the experiment was repeated 3 times. Error bars represent SD and asterisks represent significant differences (P < 0.01). Bar = 10 μm.
(TIF)

S1 Table The putative MoArk1-interacting proteins identified by Co-IP. (DOC)

S2 Table Colony diameters and conidiation of wild-type Guy11 and the Δ*Moend3* mutant. (DOC)

S3 Table Primers used in this study. (DOC)
