# Correction: MoEnd3 regulates appressorium formation and virulence through mediating endocytosis in rice blast fungus *Magnaporthe oryzae*

**DOI:** 10.1371/journal.ppat.1006554

**Published:** 2017-08-09

**Authors:** The PLOS Pathogens Staff

There are multiple errors that were introduced during the typesetting process.

“MoEnd3 is important for sexual reproduction and normal endocytosis” is incorrectly included at the end of the figure legend of Fig 1, and should be a heading in the Results section instead. The heading “MoEnd3 is important for sexual reproduction and normal endocytosis” should appear above the second paragraph of the Results section.

“MoEnd3 is involved in endocytosis of Pth11 and MoSho1” is incorrectly included at the end of the figure legend of Fig 3, and should be a heading in the Results section instead. The heading “MoEnd3 is involved in endocytosis of Pth11 and MoSho1” should appear above the third paragraph of the Results section. The publisher apologizes for the errors.

## References

[1] Li X, Gao C, Li L, Liu M, Yin Z, Zhang H, et al (2017) MoEnd3 regulates appressorium formation and virulence through mediating endocytosis in rice blast fungus *Magnaporthe oryzae*. PLoS Pathog 13(6): e1006449 https://doi.org/10.1371/journal.ppat.1006449 28628655 10.1371/journal.ppat.1006449 PMC5491321

