# Supplementary figures and images for: MoEnd3 regulates appressorium formation and virulence through mediating endocytosis in rice blast fungus Magnaporthe oryzae
Source: PLoS Pathog. 2017 Jun 19;13(6):e1006449. doi: 10.1371/journal.ppat.1006449 (PMC5491321; doi:10.1371/journal.ppat.1006449)

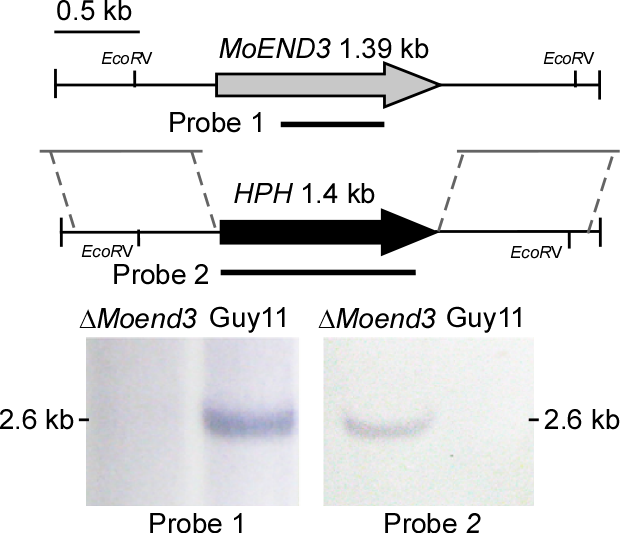

Supplement: S1 Fig — Southern blot analysis of the MoEND3 gene deletion mutants with gene specific probe (probe1) and hygromycin phosphotransferase (HPH) probe (probe2). Thick arrows indicate orientations of the MoEND3 and HPH genes. Thin lines below the arrows indicate sequence-specific gene probes. (TIF) [file ppat.1006449.s001.tif]

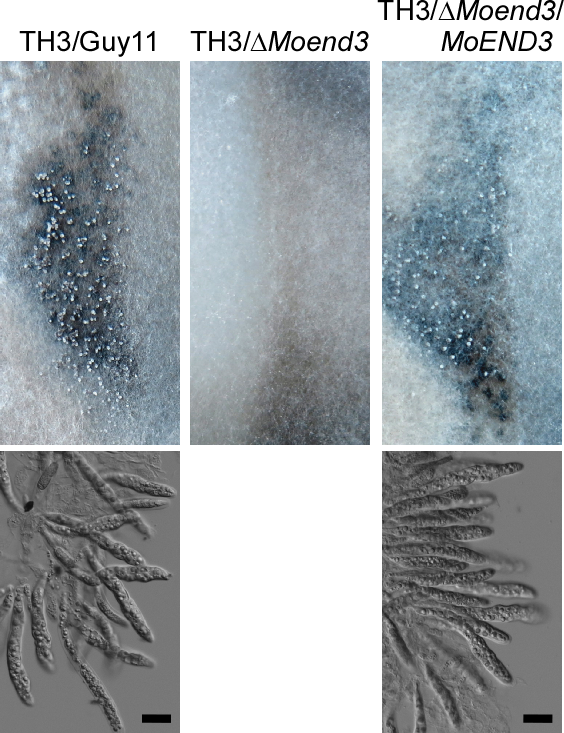

Supplement: S2 Fig — Perithecia production was photographed following three weeks of incubation. Cross between TH3 (MAT1-1) and Guy11 (MAT1-2) represents the positive control. Cross between the ΔMoend3 mutant and TH3 failed to produce peritheria or asci. Cross between the complemented strain and TH3 produced normal peritheria and asci. Bars = 20 μm. (TIF) [file ppat.1006449.s002.tif]

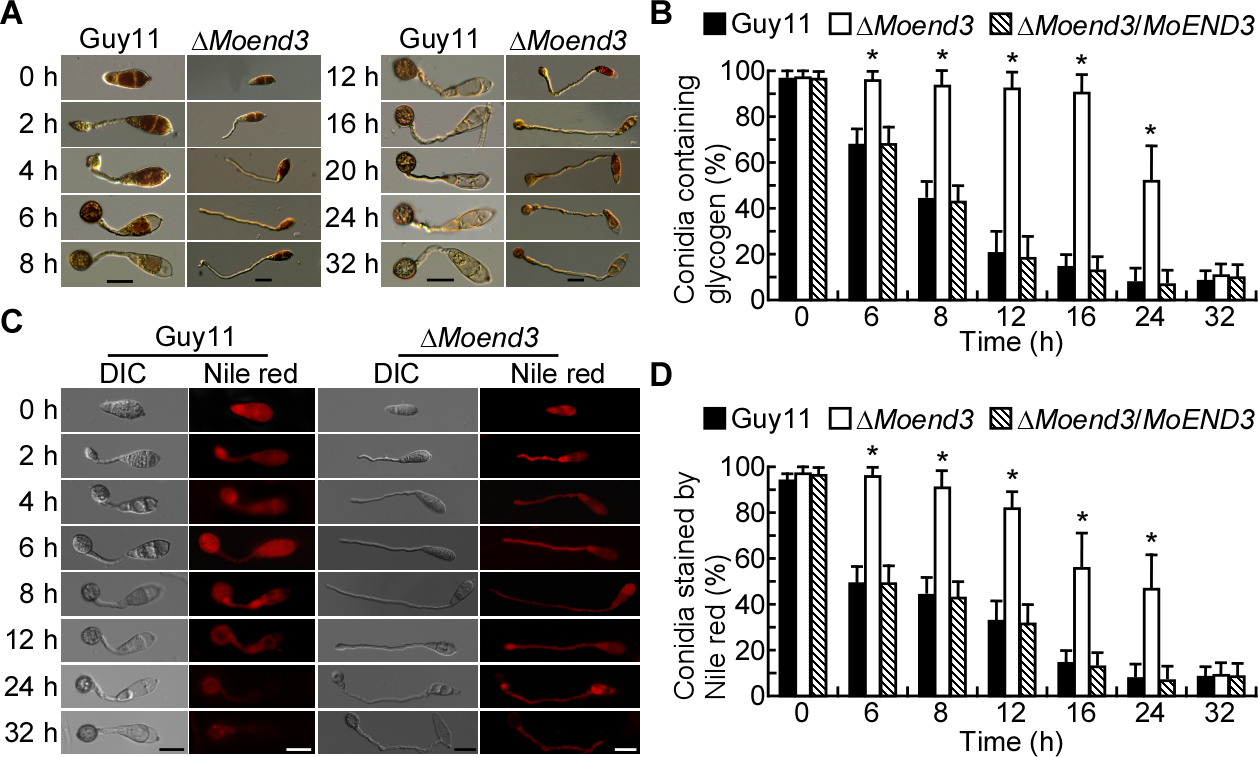

Supplement: S3 Fig — (A) Conidia were incubated on hydrophobic surface. Samples were stained with iodine solution at different time points and yellowish-brown glycogen deposits became visible immediately. Bars = 10 μm. (B) The percentage of conidia containing glycogen was recorded with observing at least 100 germinated conidia for each sample. The experiment was repeated three times. Error bars represent SD and asterisks represent significant differences (P < 0.01). (C) Conidia were allowed to germinate on hydrophobic surface. Samples were stained for the presence of lipid bodies by using Nile red. Bars = 10 μm. (D) The percentage of conidia containing abundant lipids was recorded with observing at least 100 germinated conidia. The experiment was repeated three times. The error bars represent SD and asterisks represent significant differences. (TIF) [file ppat.1006449.s003.tif]

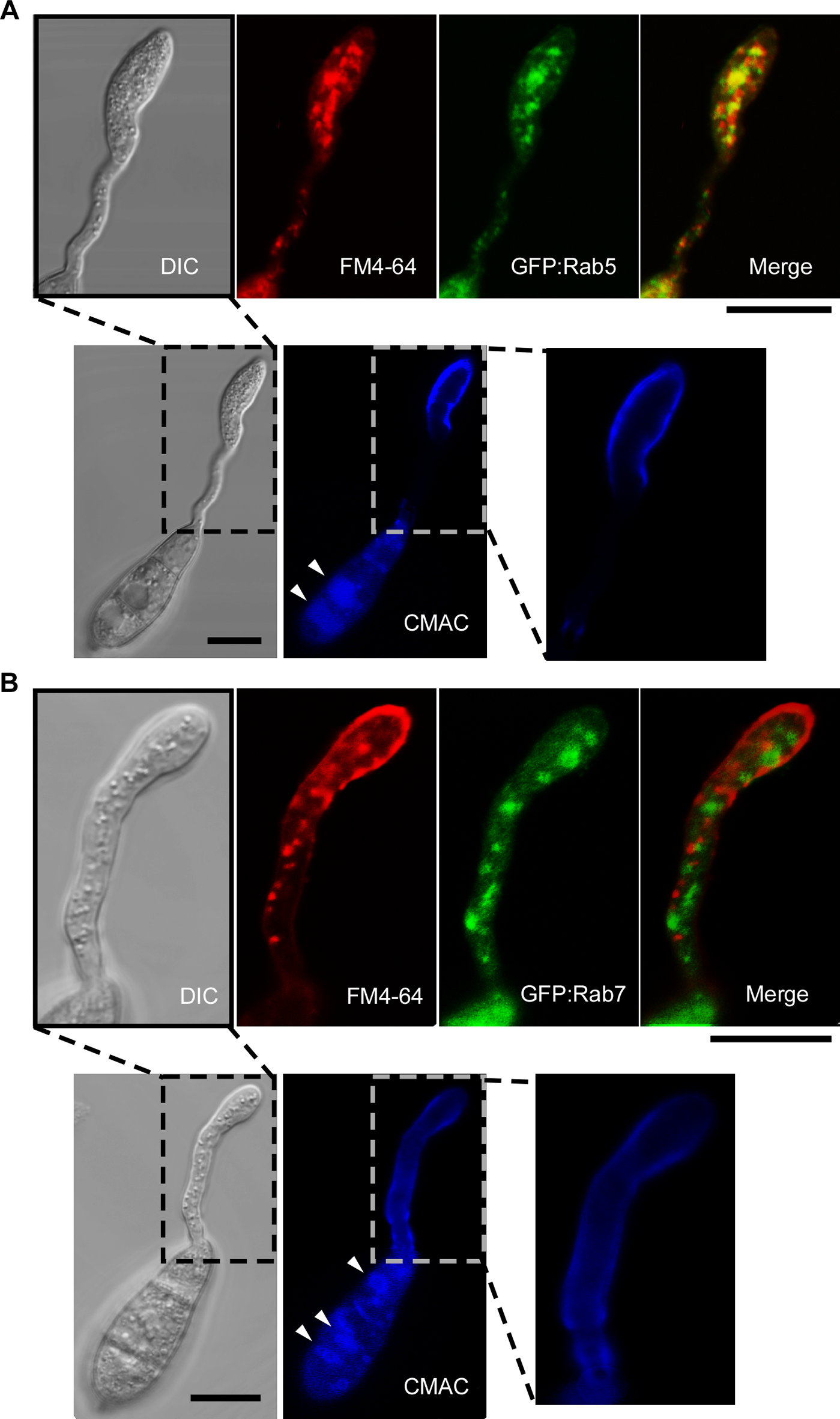

Supplement: S5 Fig — (A) Most of FM4-64 in germ tube was located to GFP:Rab5 labeled structures (early endosomes) which were distinct from CMAC stained vacuoles. (B) Co-localization of FM4-64 with GFP:Rab7 known to mark late endosomes was rarely occurred in germ tube. CMAC stained vacuoles did not appear in germ tube. (TIF) [file ppat.1006449.s005.tif]

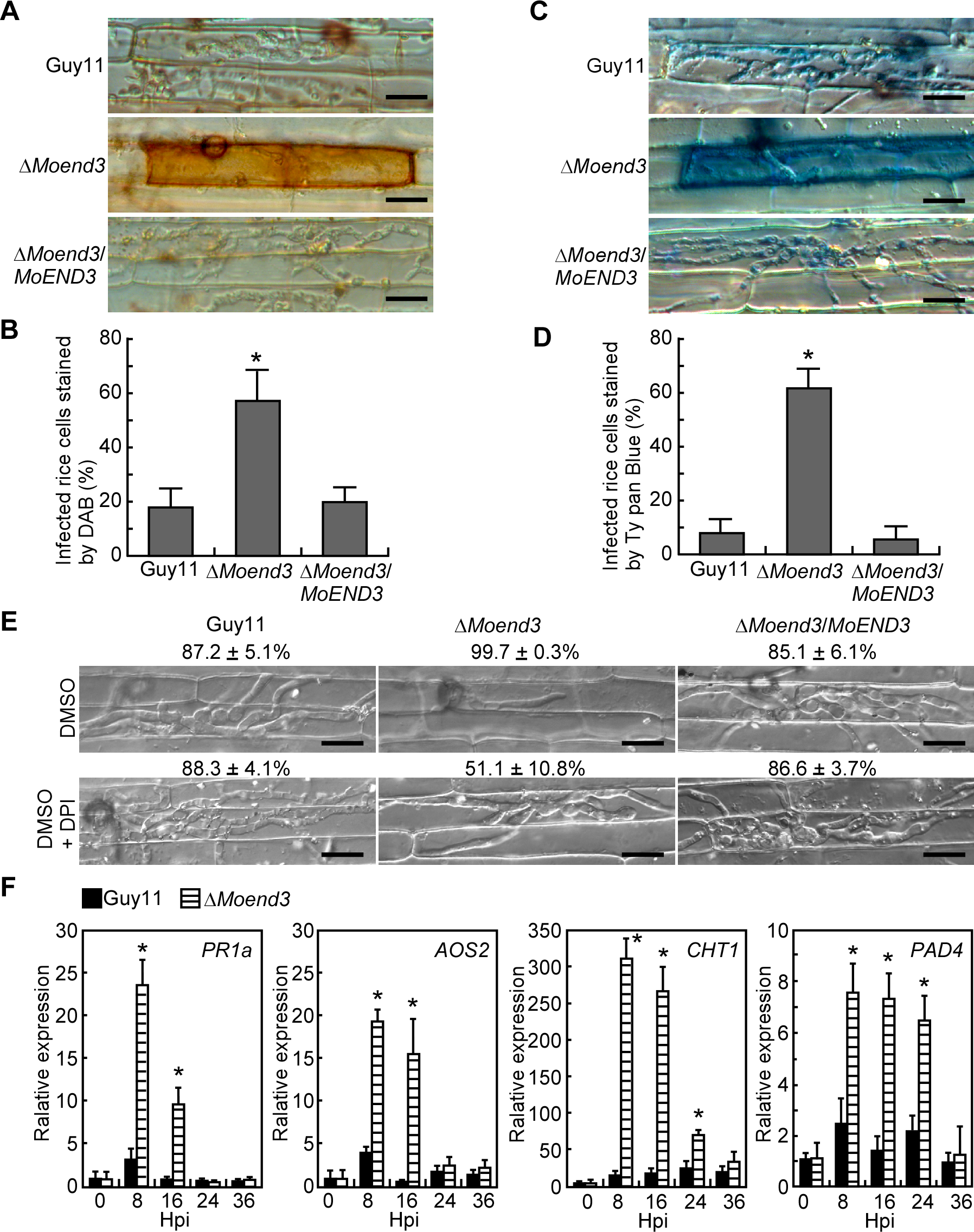

Supplement: S7 Fig — (A) Infected rice tissues were stained with DAB. DAB staining indicates that ROS accumulated in the rice cells infected by the ΔMoend3 mutant but not by Guy11 and the complemented strain at 36 hpi. Bars = 10 μm. (B) The percentage of infected rice cells stained with DAB (n = 50). Error bars represent SD and asterisk represents significant difference (P < 0.01). (C) Infected rice tissue was stained with Trypan blue. HR occurs in rice cells infected by the ΔMoend3 mutant but not Guy11 and the complemented strain. Bars = 10μm. (D) The percentage of the rice cells stained with Trypan blue (n = 50). Error bars represent SD and asterisk represents significant difference (P < 0.01). (E) IH growth in rice cells treated with DPI. When rice tissue was treated with 0.5 mM DPI dissolved in DMSO, the ΔMoend3 mutant partly restored growth in rice cells and extended IH to neighboring rice cells. The samples treated with DMSO and without DPI were used as negative controls. The percentage ± SD of the patterns showed was given. Bars = 10μm. (F) Expressions of rice pathogenesis-related genes (PR1a, AOS2, CHT1 and PAD4) were analyzed by qRT-PCR during early infection stage. RNA samples were collected from rice plants infected by Guy11 and ΔMoend3 mutant at 0, 8, 16, 24, and 36 hpi. Error bars represent the standard deviation and asterisks represent significant differences (P < 0.01). (TIF) [file ppat.1006449.s007.tif]

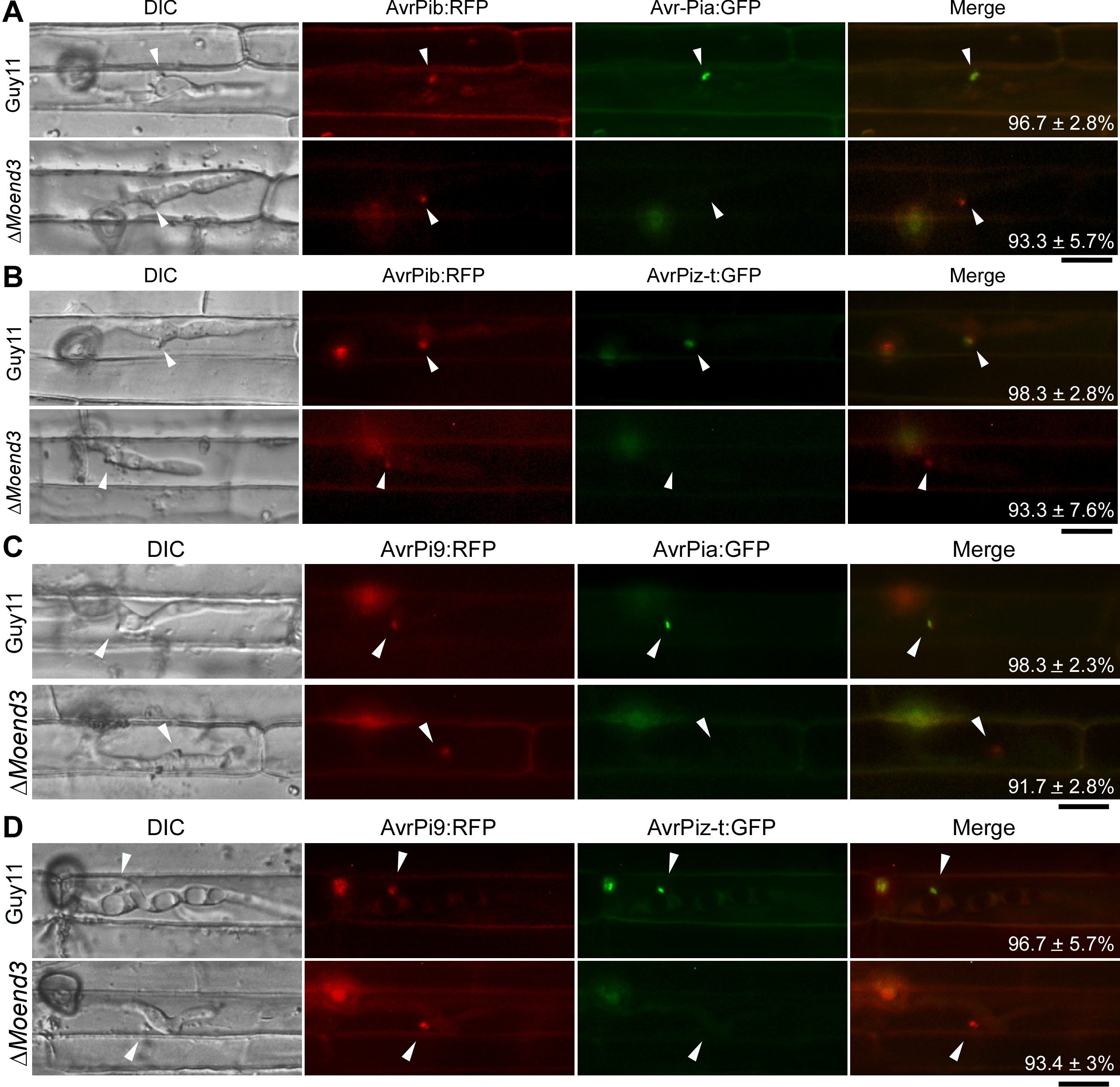

Supplement: S8 Fig — (A) Images of BICs in the rice sheath cells infected by strains expressing Avr-Pia:GFP and AvrPib:RFP. Merged images show GFP and RFP channels. White arrows indicate the BICs. The percentage ± SD (standard deviation) of the types of BIC showed was recorded from three independent experiments. In each experiment, 20 BICs containing AvrPib:RFP were observed for each strain at 24 hpi. Bar = 10 μm. (B) Images of BICs in the rice sheath cells infected by strains co-expressing AvrPiz-t:GFP and AvrPib:RFP. The percentage ± SD of the types of BIC showed was recorded from three independent experiments. In each experiment, 20 BICs containing AvrPib:RFP were observed for each strain at 24 hpi. Bar = 10 μm. (C) Images of BICs in the rice sheath cells infected by strains co-expressing Avr-Pia:GFP and AvrPi9:RFP. The percentage ± SD of the types of BIC showed was recorded from three independent experiments. In each experiment, 20 BICs containing AvrPi9:RFP were observed for each strain at 24 hpi. Bar = 10 μm. (D) Images of BICs in the rice sheath cells infected by strains co-expressing AvrPiz-t:GFP and AvrPi9:RFP. The percentage ± SD of the types of BIC showed was recorded from three independent experiments. In each experiment, 20 BICs containing AvrPi9:RFP were observed for each strain at 24 hpi. Bar = 10 μm. (TIF) [file ppat.1006449.s008.tif]

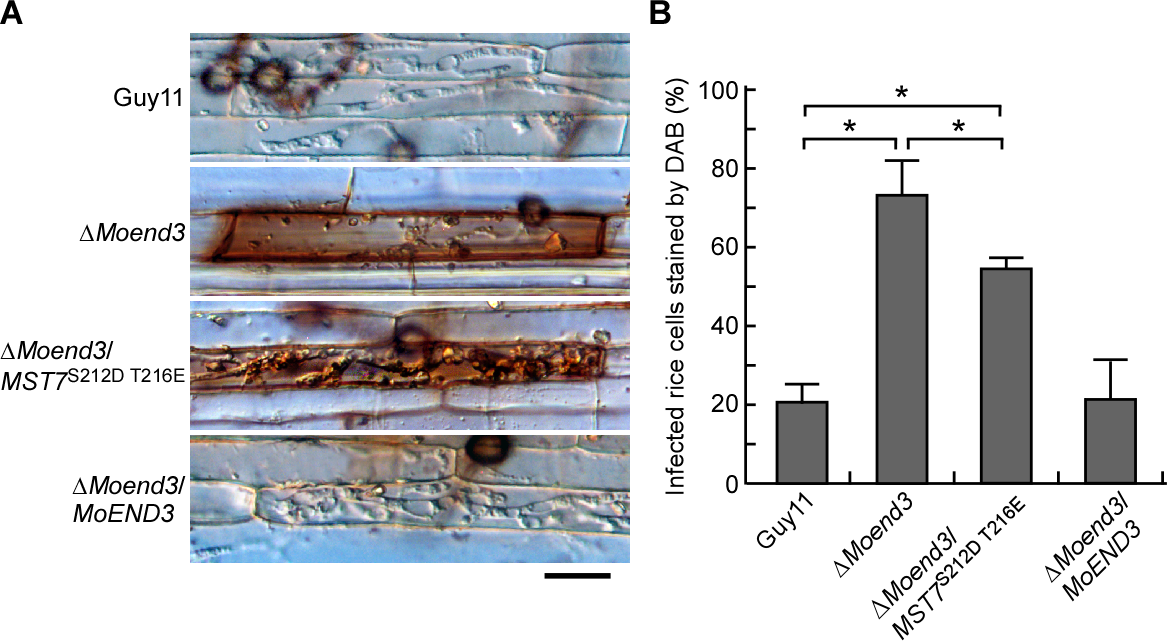

Supplement: S10 Fig — (A) DAB was used to stain ROS in the rice sheath tissue infected by Guy11, ΔMoend3, ΔMoend3/MST7S212D T216E, and ΔMoend3/MoEND3. (B) The percentage of the infected rice cells with ROS accumulation. 50 infected cells were observed for each strain and the experiment was repeated 3 times. Error bars represent SD and asterisks represent significant differences (P < 0.01). Bar = 10 μm. (TIF) [file ppat.1006449.s010.tif]
