# Supplementary material for: MoEnd3 regulates appressorium formation and virulence through mediating endocytosis in rice blast fungus Magnaporthe oryzae
Source: PLoS Pathog. 2017 Jun 19;13(6):e1006449. doi: 10.1371/journal.ppat.1006449 (PMC5491321; doi:10.1371/journal.ppat.1006449)
Supplement: S1 Table — (DOC) [file ppat.1006449.s011.doc]

| **S1 Table. The putative MoArk1-interacting proteins identified by Co-IP** | |
| --- | --- |
| Gene ID | Putative protein name |
| MGG_05193.6 | cell division cycle protein 48 |
| MGG_07768.6 | clathrin heavy chain |
| MGG_03838.6 | Ser/Thr protein phosphatase family protein |
| MGG_06358.6 | amylase-binding protein AbpA |
| MGG_03087.6 | cell division control protein 11 |
| MGG_04745.6 | ARP2/3 complex 34 kDa subunit |
| MGG_01521.6 | cell division control protein 3 |
| MGG_06649.6 | EF hand domain-containing protein |
| MGG_06180.6 | endocytosis and cytoskeletal organization protein |
| MGG_09902.6 | F-actin-capping protein subunit beta |
| MGG_08547.6 | serine/threonine-protein kinase srk1 |
| MGG_06367.6 | vesicular integral-membrane protein VIP36 |
| MGG_06361.6 | dynamin-A |
| MGG_12818.7 | F-actin-capping protein subunit alpha |
| MGG_06241.6 | vacuolar protein sorting-associated protein 21 |
| MGG_07859 | hypothetical protein |
| MGG_01569.6 | conserved hypothetical protein |
| MGG_08098.6 | conserved hypothetical protein |
| MGG_10856 | hypothetical protein |
| MGG_06958.6 | hsp70-like protein |
| MGG_11513.6 | heat shock protein SSB1 |
| MGG_06860.6 | coatomer subunit beta |
| MGG_14971.6 | elongation factor 3 |
| MGG_04719.6 | WD repeat-containing protein 38 |
