# Supplementary material for: MoEnd3 regulates appressorium formation and virulence through mediating endocytosis in rice blast fungus Magnaporthe oryzae
Source: PLoS Pathog. 2017 Jun 19;13(6):e1006449. doi: 10.1371/journal.ppat.1006449 (PMC5491321; doi:10.1371/journal.ppat.1006449)
Supplement: S2 Table — (DOC) [file ppat.1006449.s012.doc]

**S2 Table. Colony diameters and conidiation of wild-type Guy11 and the ∆*Moend3* mutant.**

| Strain | Colony Diameter (cm) | | | | Conidiation  (1x10^4^/cm^2^) |
| --- | --- | --- | --- | --- | --- |
|  | CM | MM | OM | SDC |  |
| Guy11 | 5.10 ± 0.1 | 4.20 ± 0.0 | 4.43 ± 0.1 | 3.66 ± 0.1 | 2.53 ± 0.1 |
| Δ*Moend3* | 5.13 ± 0.1 | 4.22 ± 0.1 | 4.33 ± 0.2 | 3.63 ± 0.1 | 2.6 ± 0.4 |
