# Supplementary material for: MoEnd3 regulates appressorium formation and virulence through mediating endocytosis in rice blast fungus Magnaporthe oryzae
Source: PLoS Pathog. 2017 Jun 19;13(6):e1006449. doi: 10.1371/journal.ppat.1006449 (PMC5491321; doi:10.1371/journal.ppat.1006449)
Supplement: S3 Table — (DOC) [file ppat.1006449.s013.doc]

### S3 Table. Primers used in this study.

| **Primer** | **Sequence (5’-3’)** | **Application** |
| --- | --- | --- |
| *MoEND3*-Flank F1 | GTCGACTGCTGTCGATGCAGCTAGAC | Amplifying *MoEND3* 5' flank sequence for gene knock out |
| *MoEND3*-Flank R1 | GAATTCGTGACCGTGTTGAGTTCGAG |
| *MoEND3*-Flank F2 | ACTAGTTCCTTGCTTGGCTTTAGTGG | Amplifying *MoEND3* 3' flank sequence for gene knock out |
| *MoEND3*-Flank R2 | CCGCGGCTTCGAGTCTCGCGTTCAGT |
| *MoEND3*-probeF | ACTCCTCACTCAGGAACACG | Amplifying probe 1 for southern blot and transformants screen |
| *MoEND3*-probeR | AGTCAATCTTGTTGCGCTCG |
| FL1111 | GGAGGTCAACACATCAATG | Amplifying probe 2 for southern blot |
| FL1112 | CTCTATTCCTTTGCCCTCG |
| *MoEND3*-OF | GCACACCGGAGGAATTATCT | Transformants screen |
| *HPH* comR | GCTGATCTGACCAGTTGCCTA |
| *MoEND3*-comF | ACTCACTATAGGGCGAATTGGGTACTCAAATTGGTTAAGCCTGATGAGCAGCAGCA | *MoEND3* complementation |
| *MoEND3*-comR | CACCACCCCGGTGAACAGCTCCTCGCCCTTGCTCACCCTGCCGGCCTTCTCGTCCT |
| *AOS2*-F | CAATACGTGTACTGGTCGAATGG | Amplifying *AOS2* for RT-PCR |
| *AOS2*-R | AAGGTGTCGTACCGGAGGAA |
| *CHT1*-F | CGTGGTGACCAACATCATCA | Amplifying *CHT1* for RT-PCR |
| *CHT1*-R | GAGTTGAAAGGCCTCTGGTTGT |
| *PAD4*-F | GCCAGCTCCCCTACGACTTC | Amplifying *PAD4* for RT-PCR |
| *PAD4*-R | CGTGTGCGGTGTAGGTTGTT |
| *PR1a*-F | TCTTCATCACCTGCAACTACTC | Amplifying *PR1a* for RT-PCR |
| *PR1a*-R | ATTCATCGGATTTATTCTCACC |
| FL5299 | CTTCAACACCCCTGCTATG | Amplifying the elongation factor 1a gene |
| FL5300 | CCGTTGTGGTGAATGAGTAA |
| AD-*MoEND3*-F | GAATTCATGGCACCTCGCATAGAGGC | Generating construct of AD:*MoEND3* |
| AD-*MoEND3*-R | ATCGATTCACCTGCCGGCCTTCTCGT |
| GST-*MoEND3*-F | GAATTCGTATGGCACCTCGCATAGAGGC | Generating construct of GST-MoEnd3 |
| GST-*MoEND3*-R | GCGGCCGCCCTGCCGGCCTTCTCGTCCT |
| pHZ65-*MoEND3*-F | CGACTCACTATAGGGCGAATTGGGTACTCAAATTGATGGCACCTCGCATAGAGGC | Generating construct of pHZ65:*MoEND3* |
| pHZ65-*MoEND3*-R | GCTCACCATCGTGGCGATGGAGCGCCTGCCGGCCTTCTCGTCCT |
| *MoEND3*^S222A^-R1 | GTTGCGCTCGAAGGCAGCGCGGAGAGATGCAGGGACCGTCCTGGGGATCCGG | Generating construct of *MoEND3*^S222A^:*GFP* |
| *MoEND3*^S222A^-F2 | GCATCTCTCCGCGCTGCCTTCGAGCGCAACAAGATTGACTACCAGGTTGACA |
| *MoEND3*^S222D^-R1 | GTTGCGCTCGAAGTCAGCGCGGAGAGATGCAGGGACCGTCCTGGGGATCCGG | Generating construct of *MoEND3*^S222D^:GFP |
| *MoEND3*^S222D^-F2 | GCATCTCTCCGCGCTGACTTCGAGCGCAACAAGATTGACTACCAGGTTGACA |
| RP27-*MST7*^S212D T216E^-F1 | TTTCGTAGGAACCCAATCTTCAAAATGGCCGACCCGTTTGCG | Generating construct of  *MST7*^S212D T216E^ |
| RP27-*MST7*^S212D T216E^-R1 | AAACTCATCGGCTATCTCGTTAATAAGTTCTCCCGACACA |
| RP27-*MST7*^S212D T216E^-F2 | AACGAGATAGCCGATGAGTTTGTCGGCACTTCAACTTACATGGCC |
| RP27-*MST7*^S212D T216E^-R2 | CACCACCCCGGTGAACAGCTCCTCGCCCTTGCTCACGTCCCGGATGTAAAGCCGAT |
| *H1* -F | AAGACTGAAAAGGCCGAGGCACCCGCCGCAATGGCCTCCTCCGAGGACGTCATCA | Generating construct of *H1*:*RFP* |
| *H1* -R | TTACTTGTACAGCTCGTCCATGCCGAGAGTTTAGGCGCCGGTGGAGTGGCG |
| Lifeact-F | TTTCGTAGGAACCCAATCTTCAAAATGGGTGTCGCAGATTTGATCAAGAAATTCGAAAGCATCTCAAAGGAAGAAATGGCCTCCTCCGAGGAC | Generating construct of Lifeact:RFP |
| Lifeact-R | CACCACCCCGGTGAACAGCTCCTCGCCCTTGCTCACTTAGGCGCCGGTGGAGTG |
| pHZ68-*MoARK1*-F | CGACTCACTATAGGGCGAATTGGGTACTCAAATTG CCTCATGTCTGGAGCCCGCATG | Generating construct of pHZ68:*MoARK1* |
| pHZ68-*MoARK1*-R | GTTCGGGATCTTGCAGGCCGGGCGACGTTCCCGGTCCCTCCCGC |
| BD-*MoARK1*-F | CGGGATCCTCAACGTTCCCGGTCCCTCCC | Generating construct of BD-MoArk1 |
| BD-*MoARK1*-R | CGGGATCCTCAACGTTCCCGGTCCCTCCC |
| His-*MoARK1*-F | CGGAATTCATGTTGGCGTCGGCCGCTAGG | Generating construct of His-MoArk1 |
| His-*MoARK1*-R | CGGGATCCTCAACGTTCCCGGTCCCTCCC |
| *MoARK1*-F | CTATAGGGCGAATTGGGTACTCAAATTGGTTACCTGGCTGCCTTCATCGTCATCG | Generating construct of MoArk1:3xFLAG |
| *MoARK1*-R | CTTTATAATCACCGTCATGGTCTTTGTAGTCACGTTCCCGGTCCCTCCCGC |
| BD-*MoACT1*-F | CGGGATCCATGGAAGAGGAGGTCGCCGCT | Generating construct of BD-MoAct1 |
| BD- *MoACT 1*-R | CGGAATTCTTAGAAGCACTTGCGGTGGACAATG |
| *PTH11*-F | ACTCACTATAGGGCGAATTGGGTACTCAAATTGGTTCCTAGAACCATTAGCATCTCTG | Generating construct of *PTH11*:*GFP* |
| *PTH11* -R | CTTTATAATCACCGTCATGGTCTTTGTAGTC GATGAGACCACCGGGCAGTCCT |
| *MoMSB2*-F | ACTCACTATAGGGCGAATTGGGTACTCAAATTGGTTtcctttccttcgctaccctatcc | Generating construct of *MoMSB2*:*GFP* |
| *MoMSB2*-R | CTTTATAATCACCGTCATGGTCTTTGTAGTC GTTCCAGCCGAGTGAGTTCTC |
| *MoSHO1*-F | ACTCACTATAGGGCGAATTGGGTACTCAAATTGGTTgcctctcccgacattaaccctcct | Generating construct of *MoSHO1*:*GFP* |
| *MoSHO1*-R | CTTTATAATCACCGTCATGGTCTTTGTAGTC TAGCAAAATAAGGTAGTTACT |
| *Avr-Pia-GFP-*F | TTTCGTAGGAACCCAATCTTCAAAATGCATTTTTCGACAATTTTC | Generating construct of *Avr-Pia*:*GFP* |
| *Avr-Pia-GFP-*R | CACCACCCCGGTGAACAGCTCCTCGCCCTTGCTCACGTAAGGCTCGGCAGCAAGCC |
| *AvrPiz-t-GFP-*F | TTTCGTAGGAACCCAATCTTCAAAATGCAGTTCTCAACCATCAT | Generating construct of *Avr-Piz-t*:*GFP* |
| *AvrPiz-t-GFP-*R | CACCACCCCGGTGAACAGCTCCTCGCCCTTGCTCACTTGGCGCTGAGCCTGAGGGT |
| *AvrPi9-*F | TTTCGTAGGAACCCAATCTTCAAAATGCAGTTCTCTCAGATCCT | Generating construct of *AvrPi9*:*RFP. RFP* (*AvrPi9*)-F/R were used to amplified the RFP sequence overlapped with *AvrPi9* gene sequence |
| *AvrPi9-*R | ATGACGTCCTCGGAGGAGGCCATCCAGTGCGTCTTTTCGACTT |
| *RFP*(*AvrPi9*)-F | AAGTCGAAAAGACGCACTGGATGGCCTCCTCCGAGGACGTCAT |
| *RFP*(*AvrPi9*)-R | GCGCAGAGGAGCGTGAATGTTGAGTGGAATGATTTAGGCGCCGGTGGAGTGGCGG |
| *AvrPib-*F | TTTCGTAGGAACCCAATCTTCAAAATGCGTTCCTCAACCACTTT | Generating construct of *AvrPib*:*RFP. RFP* (*AvrPib*)-F/R were used to amplified the RFP sequence overlapped with *AvrPib* gene sequence |
| *AvrPib-*R | ATGACGTCCTCGGAGGAGGCCATTTCCACGGTATATTTGCTGCC |
| *RFP*(*AvrPib*)-F | GGCAGCAAATATACCGTGGAAATGGCCTCCTCCGAGGACGTCAT |
| *RFP*(*AvrPib*)-F | GCGCAGAGGAGCGTGAATGTTGAGTGGAATGATTTAGGCGCCGGTGGAGTGGCGG |
